# OrgNet^+^: towards robust protein stability prediction with convolutional neural networks

**DOI:** 10.1093/bioinformatics/btag258

**Published:** 2026-07-07

**Authors:** Anastasia Sarycheva, Aleksandr Shumilov, Petr Popov

**Affiliations:** School of Science, Constructor University Bremen gGmbH, Bremen 28759, Germany; Constructor Labs, Bremen 28759, Germany; School of Science, Constructor University Bremen gGmbH, Bremen 28759, Germany; Constructor Labs, Bremen 28759, Germany; School of Science, Constructor University Bremen gGmbH, Bremen 28759, Germany; Constructor Labs, Bremen 28759, Germany

## Abstract

**Motivation:**

Predicting the effect of single-point mutations on protein stability is a central problem in molecular biology and protein engineering. Recent structure-based deep learning methods, particularly 3D convolutional neural networks (3D CNNs), have achieved strong predictive performance by leveraging high-resolution protein structures. However, proteins exist as heterogeneous conformational ensembles rather than single static structures, and the impact of conformational flexibility on structure-based ΔΔG predictors remains poorly characterized. Consequently, current models may yield unstable or even contradictory predictions when evaluated across alternative, yet equally plausible, conformations of the same protein.

**Results:**

We introduce OrgNet${}^{+}$, a conformational ensemble-aware and orientation-gnostic framework that explicitly incorporates protein structure flexibility during training. OrgNet${}^{+}$ is trained on augmented datasets comprising diverse conformational ensembles generated using a comprehensive set of molecular modelling methods: normal mode analysis, molecular dynamics, Monte-Carlo simulations, and a generative deep learning model. Across all ensemble types, OrgNet${}^{+}$ substantially reduces intra-ensemble prediction variance while simultaneously improving predictive accuracy. The improved performance extends to standard single-reference-structure benchmarks, even though OrgNet${}^{+}$ was trained exclusively on conformational ensembles and never exposed to the reference experimental structures.

**Availability and implementation:**

OrgNet${}^{+}$ is available at https://github.com/i-Molecule/OrgNet.

## 1 Introduction

Protein stability underpins the ability of proteins to adopt and maintain functional 3D configurations, thereby defining biological activity in both natural and engineered systems (Goldenzweig and Fleishman 2018). Single amino acid substitutions can substantially perturb this balance by altering folding energetics and structural integrity, ultimately affecting biological function (Bromberg and Rost 2009). The thermodynamic impact of such point mutations is commonly quantified as the change in Gibbs free energy change between wild-type and mutant proteins, denoted as ΔΔG (Zhang *et al.* 2012). Accurate prediction of ΔΔG is therefore central to diverse applications, including interpretation of disease-associated mutations, protein engineering, and the design of stable biotherapeutics (Zheng *et al.* 2024, Koenekoop *et al.* 2025). Because exhaustive experimental characterization of mutational effects is infeasible, reliable computational approaches for protein stability prediction are essential (Pan *et al.* 2022, Zheng *et al.* 2024).

Over the past two decades, numerous computational methods have been proposed to predict stability changes upon point mutations, ranging from physics-based energy calculations to statistical and machine-learning models (Pan *et al.* 2022). Although systematic benchmarking studies report steady progress, they also reveal persistent challenges related to dataset bias, limited generalization, and diminishing performance gains among newly proposed methods (Diaz *et al.* 2024). Across comparative evaluations, structure-based approaches consistently outperform sequence-only methods, highlighting the importance of local 3D environments and long-range interactions in determining mutation effects (Blaabjerg *et al.* 2023, Zheng *et al.* 2024). By explicitly encoding spatial context, structure-based models are more closely grounded in the physical determinants of protein stability (Li *et al.* 2020, 2024). However, this increased physical fidelity may come at the cost of sensitivity to the input protein structure, particularly for models that operate directly and solely on 3D representations. In particular, 3D convolutional neural networks (3D CNNs) are sensitive to the spatial orientation of the input protein, leading to inconsistent predictions for the same structures presented in different orientations. Recently, we addressed this limitation by developing OrgNet, an orientation-gnostic 3D CNN for protein stability prediction, and demonstrated its superior performance compared to Special Euclidean Group in 3 dimensions (SE(3))-equivariant 3D CNN architectures (Buyanov *et al.* 2025). OrgNet eliminates sensitivity to arbitrary rigid-body rotations through explicit orientation standardization of the local structural environment. As a result, OrgNet produces consistent ΔΔG predictions under arbitrary protein input orientations and achieves state-of-the-art performance among structure-based methods. Yet another important source of prediction uncertainty remains largely unaddressed in modern learning-based protein stability prediction: protein structure flexibility.

From a physics-based perspective, accurate modelling of mutation-induced stability changes should capture structural rearrangements caused by a point mutation and the associated change in the free energy of the folded state (Kellogg *et al.* 2011). Arguably, free energy perturbation approach is one of the most physically grounded to satisfy these requirements by explicitly modelling the chemical transformation induced by a mutation while performing extensive conformational sampling to estimate free-energy differences (Koenekoop *et al.* 2025). However, such approaches are computationally expensive, technically demanding, and difficult to deploy at scale. On the other hand, most widely used structure-based machine-learning methods operate on static structural representations derived from a single reference conformation, completely ignoring protein structure flexibility. A few exceptions include SDM, which uses conformationally constrained environment-specific substitution tables, evaluating mutations across nine conformations (Pandurangan *et al.* 2017), and normal mode analysis (NMA) based methods, such as ENCoM (Frappier *et al.* 2015) and DynaMut (Rodrigues *et al.* 2018, 2021). ENCoM (Elastic Network Contact Model) uses NMA to model mutation-induced conformational fluctuations, estimating ΔΔG from differences in vibrational entropy between wild-type and mutant harmonic ensembles. Building on this idea, DynaMut incorporates NMA-derived features into Random Forest predictors (Rodrigues *et al.* 2018, 2021), reporting performance gains over a broad range of structure-based predictors. Nevertheless, those approaches remain limited to NMA-driven deformations of the reference structure, thus, do not reflect other types of biologically plausible conformational ensembles. Moreover, to the best of our knowledge, none of the deep learning methods explicitly address protein structure flexibility, including state-of-the-art CNN-based approaches. As a result, the robustness of these methods to biologically realistic flexible deformations remains largely unexplored.

**Figure 1:**
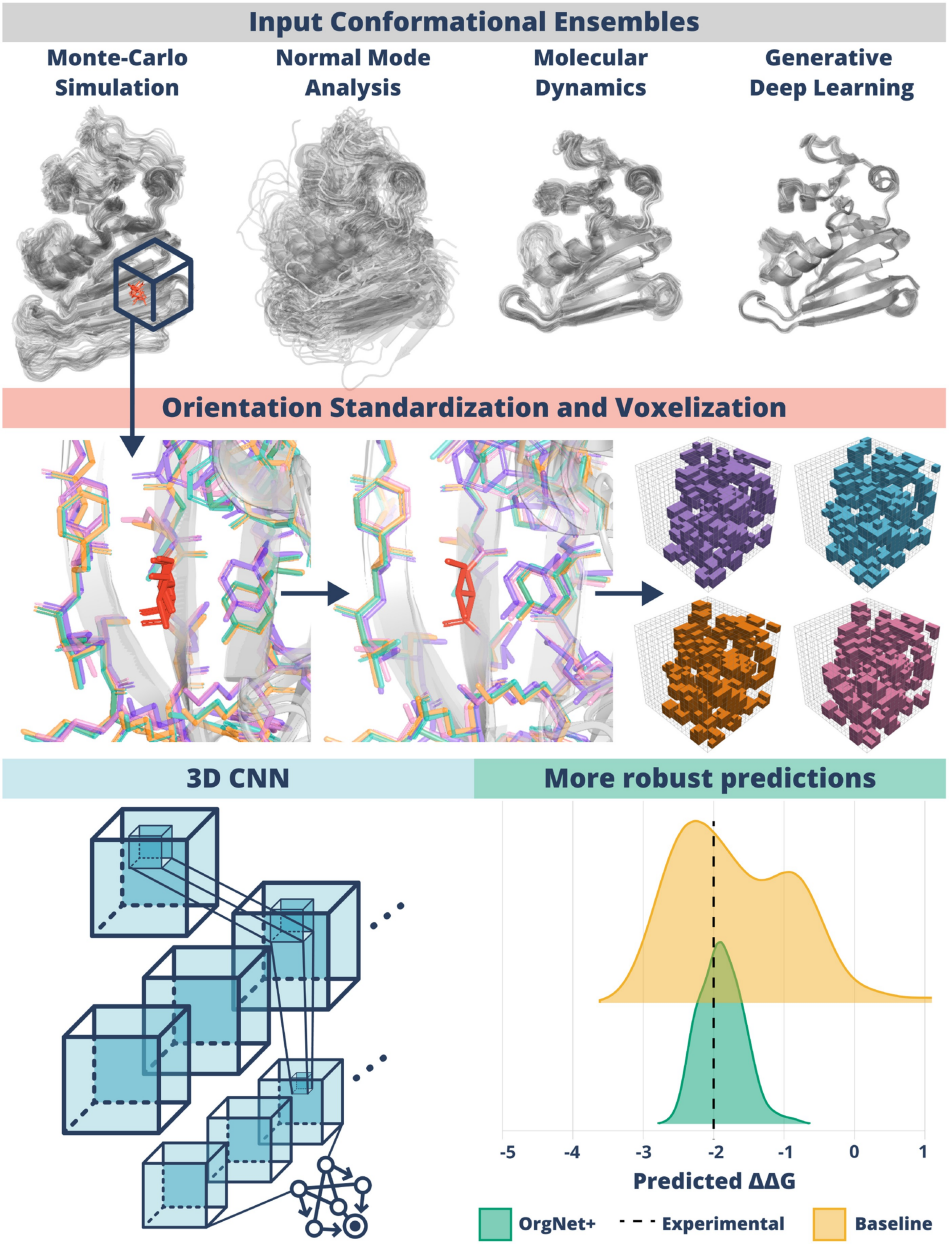
Schematic illustration of the OrgNet${}^{+}$ approach. Conformational ensembles generated with multiple modelling approaches are processed by an orientation standardization given a point mutation. This is followed by the voxel grids computations, which are fed into a 3D convolutional neural network. The OrgNet${}^{+}$ approach promotes more consistent ΔΔG predictions across conformational ensembles, reducing prediction variance relative to the baseline OrgNet approach trained on the static protein structures.

With the advances of molecular modelling and conformational ensemble generation, it becomes possible to enhance the training datasets and test benchmarks with data augmentation technique. We hypothesize that training structure-based predictors on multiple, slightly different protein conformations for the given point mutations yields more robust predictive models for ΔΔG. In this work, we investigate how conformational ensembles generated by diverse modelling approaches, including physics-based and deep learning approaches, affect robustness and consistency of 3D CNN predictors. We introduce OrgNet${}^{+}$, extending the OrgNet architecture to explicitly learn stable ΔΔG predictions across alternative conformations of the same protein (Fig. 1), a setting not addressed by prior methods. By explicitly exposing the model to controlled structural variability during training, OrgNet${}^{+}$ substantially reduces prediction variance without compromising accuracy, outperforming both the original OrgNet and other state-of-the-art structure-based predictors.

## 2 Materials and methods

### 2.1 Conformational ensemble generation

We used the S2648 dataset (Dehouck *et al.* 2011) for training, which comprises experimentally measured single-point mutations across 131 proteins, and performed five-fold cross-validation using homology-based splits (Savojardo *et al.* 2016). As the test set, we used the S669 benchmark (Pancotti *et al.* 2022), which contains point mutations across 94 proteins and was explicitly constructed to share less than 25% sequence identity with S2648. While S669 is a standard test benchmark, recent study reported incorrect entries and mutations affecting protein–protein or protein–ligand interactions (Hernández *et al.* 2023), also providing a curated subset of S669—the S461 dataset. Therefore, we additionally report results on the S461 dataset, which comprises point mutations across 48 proteins. To enforce prediction symmetry, each point mutation in all datasets was complemented with the corresponding reverse mutation.

For each wild-type protein structure in the S2648 and S669 datasets, we generated conformational ensembles of 100 structures using the methods described below (see Table 1, available as supplementary data at *Bioinformatics* online, for computational cost details).

#### 2.1.1 Normal modes

iMod (Lopéz-Blanco *et al.* 2011) performs NMA in internal coordinate space to model collective protein motions. For each protein structure, we computed the 10 lowest-frequency modes using the iMODE/iMC tools from the iMOD suite. Conformational sampling was performed with iMC, which generates trial moves by randomly selecting a low-frequency mode and an associated amplitude, and accepts/rejects moves with a Metropolis criterion based on the change in harmonic energy at temperature T=350 K.

The flexible deformation of a protein structure can be written as a linear combination of normal modes:


(1) $$\Delta r(a)=\sum_{k=1}^{10}a_{k}u_{k},$$

where $u_{k}$ is mode *k*, $a_{k}$ is the corresponding amplitude, and $a={\left\{a_{k}\right\}}_{k=1}^{10}$ is the vector of the amplitudes. The root mean square deviation (RMSD) with respect to the starting structure induced by a given amplitude vector a is then:


(2) $$\text{RMSD}(a)=\sqrt{\frac{1}{N}\sum_{i=1}^{N}{\left\|\Delta r_{i}(a)\right\|}^{2}},\;$$

where $\Delta r_{i}(a)$ is the deviation of the *i*th atom, and *N* is the total number of atoms. To control the RMSD value, we scanned a single amplitude scalar *a* over a grid from 1 to 17 with the step of 0.5; for each value of *a*, iMC sampled mode coefficients $a_{k}$ while using *a* to set the overall scale of the perturbations, and we computed the RMSD distribution of the resulting conformations relative to the starting structure. We then selected, for each protein, the amplitude *a* that produced an RMSD distribution centred around approximately 3 Å.

#### 2.1.2 Non-linear NMA

NOLB (Hoffmann and Grudinin 2017) extends classical NMA by introducing non-linear deformations by means of rigid-body motions of residue blocks. For each protein structure, we computed the 10 lowest-frequency normal modes using the NOLB software. Conformations were generated by sampling linear combinations of these modes and controlling the RMSD value relative to the reference structure (Popov and Grudinin 2015). Specifically, for each sample, we first drew a random set of mode coefficients in a range from [−1,1], computed the resulting RMSD to the reference structure, and then rescaled all the coefficients so that the final deformed structure matched a target RMSD of 3.0 Å.

#### 2.1.3 Rosetta Backrub

Rosetta Backrub protocol (Lauck *et al.* 2010) is another modelling approach, which samples concerted backbone rotations around short residue segments while preserving the global fold of the input protein structure. We sampled 100 conformations using the Rosetta Backrub tool with the following parameters: effective temperature for Monte-Carlo sampling of 2.0, side-chain conformation repacking probability of 0.5, and conformations were saved every 1500 Monte-Carlo steps.

#### 2.1.4 Molecular dynamics

All-atom molecular dynamics (MD) simulations were performed using GROMACS (Abraham *et al.* 2015). Proteins were solvated using the TIP3P water model with a 2.0 nm padding and an ionic strength of 0.15 M, and parameterized using the amber14sb force field. Following energy minimization, systems were equilibrated for 1000 ps in the NVT (constant volume and temperature) ensemble and 1000 ps in the NPT (constant pressure and temperature) ensemble using the v-rescale thermostat (target T=300 K; $\tau_{t}=0.1$ ps). During NPT equilibration, pressure was maintained at 1.0 bar using the C-rescale barostat with isotropic coupling ($\tau_{p}=2.0$ ps). Production simulations were run for 10 ns with a 2 fs integration time step, using the same thermostat ($\tau_{t}=0.5$ ps) and barostat (1.0 bar, isotropic; $\tau_{p}=5.0$ ps) (see Table 2, available as supplementary data at *Bioinformatics* online, for more details). Conformational ensembles were constructed by extracting 100 snapshots at regular intervals of 100 ps from the production trajectories.

#### 2.1.5 Boltz-2

Boltz-2 employs an SE(3)-equivariant generative architecture that enables sampling diverse conformations conditioned on the input multiple sequence alignment (MSA) for the target protein sequence (Passaro *et al.* 2025). For each target protein sequence, we generated MSAs using the ColabFold pipeline (Mirdita *et al.* 2022). Ensembles were generated via diffusion-based sampling with 200 denoising steps per prediction and a single independent diffusion sample. Iterative refinement was performed using three recycling iterations. All other inference parameters were kept at their default values, including the diffusion step scale of 1.638. Due to GPU memory limitations, Boltz-2 failed to generate conformations for the following proteins: 1KFW, 1TYV, 3GLY from S2648; and 1BA3, 1PRE, 2JIE from S669.

### 2.2 Orientation and voxel generation

Each point mutation was processed using the orientation standardization and voxelization pipeline described in detail in the original OrgNet paper (Buyanov *et al.* 2025). Briefly, for a mutation site residue *k*, a local residue-centred coordinate frame is defined based on the backbone atoms’ position, and the input structure is re-oriented to be aligned with the standardized coordinate frame. Following re-orientation, each structure is voxelized into a 16×16×16 grid centred at the $C_{\beta }$ atom of the mutation site residue. The voxel grid encodes seven physicochemical channels—hydrophobicity, hydrogen bond acceptor capacity, hydrogen bond donor capacity, aromaticity, positive ionizability, negative ionizability, and occupancy—computed using the HTMD library (Doerr *et al.* 2016). Given voxel grids computed for the wild-type [(WT) and mutant (MT)] structures, the final point mutation representation was constructed by concatenating the [WT] grid and the difference grid, resulting in a combined representation [WT, WT–MT].

### 2.3 Model architecture and training

We used the OrgNet 3D CNN architecture, comprising an input 3D convolutional layer with 16 channels, intermediate convolutional blocks with 80 and 400 channels and rectified linear unit (ReLU) activations followed by max-pooling, and an output block with a 512-channel 3D convolution feeding into a two-layer fully connected regression head (512 and 128 units) with Gaussian error linear unit (GELU) activations (Buyanov *et al.* 2025). The final OrgNet${}^{+}$ model was trained on the S2648 dataset augmented with all generated conformational ensembles. For the ablation studies, separate models were trained using the augmented datasets corresponding to the specific conformational ensemble generation methods. All hyperparameters followed the OrgNet configuration, except for increased batch sizes (16 384 for full-ensemble training and 4096 for single-ensemble experiments). Final predictions were obtained by averaging outputs from five models, each selected based on the best validation loss in its respective fold from five-fold cross-validation.

All computations were performed on an Ubuntu 22.04.5 LTS system equipped with an Intel Xeon w7-3455 CPU (24 cores), 251 GB RAM, and an NVIDIA GPU (CUDA 12.4). The software environment included the CUDA toolkit 12.4 and PyTorch 2.7.1+cu126.

### 2.4 Voxel-based structural similarity

To quantify variability in the augmented voxel representations, we computed a 3D structural similarity index (3D-SSIM). SSIM is a perceptual similarity metric originally developed for image analysis to capture structural patterns rather than point-wise differences, such as in root mean square error (RMSE) or mean absolute error (MAE) (Wang *et al.* 2004). For two signals *x* and *y*, SSIM is defined as


(3) $$\text{SSIM}(x,y)=\frac{\left(2\mu_{x}\mu_{y}+C_{1})(2\sigma_{xy}+C_{2}\right)}{\left(\mu_{x}^{2}+\mu_{y}^{2}+C_{1})(\sigma_{x}^{2}+\sigma_{y}^{2}+C_{2}\right)},$$

where $\mu_{x}$ and $\mu_{y}$ denote local means, $\sigma_{x}^{2}$ and $\sigma_{y}^{2}$ local variances, $\sigma_{xy}$ the covariance, and $C_{1}$ and $C_{2}$ are small stabilization constants that prevent numerical instabilities in regions of low intensity or variance. Following the original SSIM formulation (Wang *et al.* 2004), we define $C_{1}={\left(K_{1}\cdot R\right)}^{2}$ and $C_{2}={\left(K_{2}\cdot R\right)}^{2}$, where *R* denotes the dynamic range of voxel intensities and $K_{1}=0.01$, $K_{2}=0.03$. Local statistics were computed over local spatial neighborhoods. This formulation can be extended to volumetric data by computing SSIM over local cubic neighborhoods within 3D voxel grids. Specifically, we used Gaussian-weighted cubic neighborhoods, where local means, variances, and covariances were estimated using a normalized 3D Gaussian kernel with standard deviation σ=1.5 to match the reference SSIM implementation (Wang *et al.* 2004). The resulting voxel-wise 3D-SSIM map was cropped near the boundaries to avoid edge effects and then averaged over the full voxel volume to obtain a single similarity score for a pair of voxel representations of a given point mutation. We computed 3D-SSIM using the occupancy channel only, as it provides a dense representation of all atoms in the local environment, in contrast to the sparse atom-specific channels. For a given point mutation, we considered a set of voxel grids $V_{i}(i=1,\ldots ,N)$ corresponding to a conformational ensemble of N=100 structures and computed the 3D-SSIM score for all pairs of conformations. The conformational ensemble-averaged 3D-SSIM score was defined as the mean of the pairwise 3D-SSIM values across all pairs:


(4) $$\bar{3D-\text{SSIM}}=\frac{1}{\frac{N(N-1)}{2}}\sum_{i=1}^{N-1}\sum_{j=i+1}^{N}3D-\text{SSIM}(V_{i},V_{j}).$$

When considering the full dataset, each point mutation is associated with five independent conformational ensembles. In this case, the structural similarity score for a mutation was obtained by averaging $\bar{3D-\text{SSIM}}$ across the five ensembles.

To assess the relationship between voxel-based variability and prediction robustness, we computed the Pearson correlation coefficient between $\bar{3D-\text{SSIM}}$ and the corresponding prediction uncertainty $\sigma_{\text{pred}}$ across point mutations. Since smaller $\bar{3D-\text{SSIM}}$ values indicate lower similarity within a set of voxel grids, one would expect a negative correlation, that is, increased structural diversity leads to higher prediction uncertainty. The prediction uncertainty for a point mutation, in turn, was defined as the standard deviation of predictions across the conformational ensemble:


(5) $$\sigma_{\text{pred}}=\sqrt{\frac{1}{N-1}\sum_{i=1}^{N}{\left({\hat{y}}_{i}-\bar{\hat{y}}\right)}^{2}},$$

where ${\hat{y}}_{i}$ is the model’s prediction for voxel grid $V_{i}$ and $\bar{\hat{y}}$ denotes the mean predicted ΔΔG across the N=100 conformations in the ensemble. For the full dataset, prediction uncertainty was computed analogously using all conformations from the five ensembles (N=500).

## 3 Results

### 3.1 Sensitivity of 3D CNN approaches to protein structure flexibility

**Figure 2:**
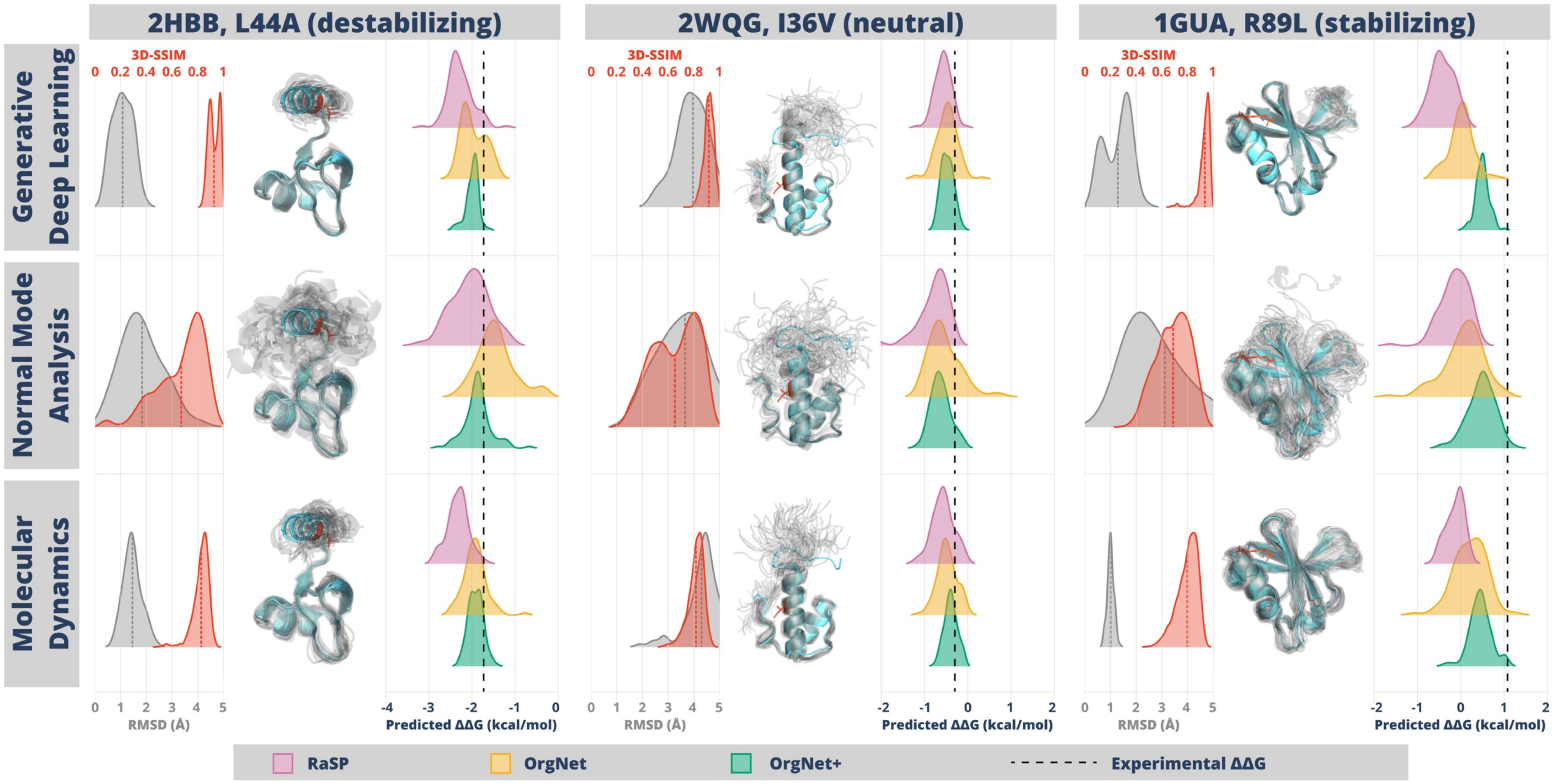
Illustration of the sensitivity problem of 3D CNN-based ΔΔG predictors to protein conformational flexibility for representative stabilizing, neutral, and destabilizing point mutations. Conformational ensembles were generated using molecular dynamics, normal mode analysis, and generative deep learning. The reference structure and generated conformations are shown with light-blue and grey cartoon representations, respectively; the point mutation residue is shown with red sticks representation. Protein structures are accompanied by distributions of global (RMSD, grey) and local (mean pairwise 3D-SSIM, red) structural similarity. Predicted ΔΔG values across the conformational ensembles are shown for RaSP, OrgNet, and OrgNet${}^{+}$ (pink (top), yellow (middle), and green (bottom) distributions, respectively), and the experimental ΔΔG value is shown with a vertical black dash line. While OrgNet and RaSP exhibit substantial intra-ensemble prediction variability, OrgNet${}^{+}$ consistently produces tighter ΔΔG distributions across all ensemble types.

To illustrate the sensitivity problem of structure-based methods, specifically those based on 3D CNN, with respect to the protein flexible deformations, we analyzed representative point mutations evaluated across conformational ensembles generated using diverse sampling strategies, including NMA, MD, Monte-Carlo simulations, and a generative deep learning model (see Section 2.1). For each conformation, ΔΔG predictions were computed using OrgNet (Buyanov *et al.* 2025) and RaSP (Blaabjerg *et al.* 2023), state-of-the-art structure-based 3D CNN methods. Figure 2 shows the distribution of the predicted ΔΔG values across conformational ensembles for representative destabilizing, neutral, and stabilizing mutations, together with the corresponding global and local structural variability quantified by RMSD and 3D-SSIM, respectively. Both OrgNet and RaSP exhibit substantial intra-ensemble prediction spread, that can even span across 0, thus, predicting opposite stability effects for the same point mutation.

Notably, although OrgNet explicitly resolves SE(3) variance in 3D CNN-based ΔΔG prediction through orientation standardization (Buyanov *et al.* 2025), it remains sensitive to structural variability arising from flexible deformations. Thus, protein structure flexibility is a major and currently unresolved source of uncertainty for structure-based ΔΔG predictors.

### 3.2 The OrgNet${}^{+}$ approach

Motivated by the observed sensitivity to protein structure flexibility, we hypothesized that explicitly exposing the model to alternative plausible conformations during training would improve model’s robustness. To test this hypothesis, we generated augmented training dataset by constructing conformational ensembles using a diverse set of molecular modelling approaches: Monte-Carlo simulations with Rosetta Backrub (Lauck *et al.* 2010), MD with Gromacs (Abraham *et al.* 2015), NMA with iMod (Lopéz-Blanco *et al.* 2011), non-linear NMA with NOLB (Hoffmann and Grudinin 2017), and generative deep learning with Boltz-2 (Passaro *et al.* 2025) (Fig. 1). Namely, for each protein structure in the original dataset, we modelled 100 alternative conformations per molecular modelling approach, thus each point mutation is represented by 500 rather than a single static structure. We used the OrgNet protocol (Buyanov *et al.* 2025) to train the models, as OrgNet is already robust with respect to the rigid-body orientations of the input. No architectural modifications were introduced; instead, robustness to flexibility was learned implicitly by minimizing the prediction error across structurally perturbed inputs sharing the same experimental ΔΔG. Final predictions were obtained by aggregating outputs from independently trained models using five-fold cross-validation. We refer to models trained on these augmented datasets as OrgNet${}^{+}$.

When evaluated on unseen conformational ensembles, OrgNet${}^{+}$ produced markedly tighter ΔΔG distributions across all ensemble types compared to both OrgNet and RaSP (Fig. 2). This effect was observed across destabilizing, neutral, and stabilizing mutations and all types of conformational ensembles. More specifically, OrgNet${}^{+}$ substantially reduced intra-ensemble prediction uncertainty. On the full augmented S669 dataset, the average prediction standard deviation $\sigma_{\text{pred}}$ decreased from 0.57±0.23 for OrgNet to 0.42±0.13 for OrgNet${}^{+}$ for the direct mutations, with a similar robustness gain for the reverse mutations (see Table 1). A comparable trend was observed on S461, where $\sigma_{\text{pred}}$ decreased from 0.58±0.25 to 0.42±0.14. This performance gain was consistent across individual ensemble types (see Tables 4 and 5, available as supplementary data at *Bioinformatics* online), including more diverse conformational ensembles obtained with NOLB and Backrub, which exhibit the lowest ensemble-averaged structural similarity (see Table 3, available as supplementary data at *Bioinformatics* online): e.g. NOLB ensembles show mean $\bar{3D-\text{SSIM}}$ values of 0.42 and 0.44 for S669 and S461, respectively, whereas Boltz-2 ensembles are substantially more homogeneous ($\bar{3D-\text{SSIM}}\approx 0.87$). Importantly, reduced prediction variance did not come at the expense of accuracy. On the contrary, OrgNet${}^{+}$ simultaneously improved predictive performance, achieving lower RMSE and higher Pearson correlation than OrgNet on both test sets (see Table 1). For instance, on S669 (direct setting), RMSE decreased from 1.66 (OrgNet) to 1.54 (OrgNet${}^{+}$), while Pearson *r* increased from 0.31 to 0.41. Similarly, on S461, OrgNet${}^{+}$ achieved the lowest RMSE (1.15) and highest Pearson correlation (r=0.54).

**Table 1:** Performance metrics for direct and reverse mutations for the augmented test S669 and S461 datasets comprising all five conformational ensembles, including prediction accuracy (RMSE, MAE, Pearson *r*), prediction uncertainty ($\sigma_{\text{pred}}$) due to conformational flexibility, and the correlation between prediction uncertainty ($\sigma_{\text{pred}}$) and conformational flexibility expressed by $\bar{3D-\text{SSIM}}$.^a^

Test	Method	Direct					Reverse				
		RMSE	MAE	*r*	$\sigma_{\text{pred}}$	$r_{\bar{3D-\text{SSIM}}-\sigma_{\text{pred}}}$	RMSE	MAE	*r*	$\sigma_{\text{pred}}$	$r_{\bar{3D-\text{SSIM}}-\sigma_{\text{pred}}}$
S669	RaSP	1.60	1.13	0.37	0.474 ± 0.278	−0.29	1.97	1.50	0.26	0.400 ± 0.209	−0.30
S669	OrgNet	1.66	1.22	0.31	0.570 ± 0.225	−0.29	1.68	1.24	0.31	0.593 ± 0.237	−0.27
S669	OrgNet${}^{+}$	**1.54**	**1.13**	**0.41**	0.420 ± 0.130	**−0.41**	**1.56**	**1.14**	**0.40**	0.430 ± 0.134	**−0.39**
S461	RaSP	1.17	0.84	0.52	0.474 ± 0.283	−0.31	1.73	1.34	0.30	0.381 ± 0.199	−0.33
S461	OrgNet	1.32	0.99	0.43	0.576 ± 0.245	−0.32	1.34	1.01	0.44	0.595 ± 0.256	−0.27
S461	OrgNet${}^{+}$	**1.15**	**0.88**	**0.54**	0.418 ± 0.137	**−0.42**	**1.18**	**0.89**	**0.53**	0.426 ± 0.140	**−0.37**

a Best values for each performance metric are highlighted in bold.

### 3.3 Comparison with state-of-the-art structure-based predictors

We next evaluated OrgNet${}^{+}$ against OrgNet and RaSP on the S669 and S461 test benchmarks. Performance was assessed for individual ensembles (see Tables 4 and 5, available as supplementary data at *Bioinformatics* online) as well as on the combination of all five ensembles (see Table 1). Across all evaluation settings, OrgNet${}^{+}$ consistently outperformed both OrgNet and RaSP in terms of prediction accuracy and robustness. On S669 in the direct mutation setting, OrgNet${}^{+}$ achieved an RMSE of 1.54 and MAE of 1.13, compared to 1.66/1.22 for OrgNet and 1.60/1.13 for RaSP, while also achieving the highest Pearson correlation (r=0.41; see Table 1). A similar improvement was observed for reverse mutations, where OrgNet${}^{+}$ maintained balanced performance (RMSE 1.56, r=0.40). On the S461 benchmark, OrgNet${}^{+}$ also achieved the best overall performance, with RMSE values of 1.15 (direct) and 1.18 (reverse), outperforming both OrgNet and RaSP (see Table 1). Note that RaSP demonstrated poor performance on the reverse mutations, most likely due to the one-sided training strategy, which does not explicitly include reverse mutations in the training (Blaabjerg *et al.* 2023). In contrast, both OrgNet and OrgNet${}^{+}$ maintained balanced performance with respect to direct and reverse point mutations. OrgNet${}^{+}$ demonstrates improved accuracy and robustness compared to OrgNet with respect to both surface and buried residues (see Fig. 2 and Table 14, available as supplementary data at *Bioinformatics* online). For instance, the robustness gains ($\Delta \sigma_{\text{pred}}$) over OrgNet are 0.226/0.101 (S669) and 0.250/0.103 (S461) for buried/surface residues using a standard solvent-accessible surface area (SASA) cut-off of 0.2 (Chen *et al.* 2020). We also evaluated OrgNet${}^{+}$ on the original S669 and S461 benchmarks of single reference structures only, i.e. without conformational ensemble augmentation. Despite being trained on exclusively augmented data without reference structures, OrgNet${}^{+}$ consistently outperformed OrgNet and remained competitive with the best-performing structure-based predictors (Table 2). On S669, OrgNet${}^{+}$ achieved a Pearson correlation of 0.44 with an RMSE of 1.50 and MAE of 1.10 in the direct mutation setting, improving upon OrgNet (RMSE 1.55, MAE 1.12) and RaSP (RMSE 1.62). Importantly, OrgNet${}^{+}$ maintained balanced performance between direct and reverse mutations (RMSE 1.52 in reverse), whereas several competing methods exhibited substantial degradation on reverse mutations. On the curated S461 benchmark, OrgNet${}^{+}$ also improved performance, achieving r=0.58 with RMSE 1.08 and MAE 0.84 for direct mutations, compared to OrgNet (r=0.54, RMSE 1.16). While a small number of methods marginally outperform OrgNet${}^{+}$ in individual metrics on S461, OrgNet${}^{+}$ remains among the top-performing models overall and exhibits robust, symmetric performance across direct and reverse settings. These results demonstrate that ensemble-aware training improves robustness to conformational variability without compromising accuracy on standard single-structure benchmarks.

**Table 2:** Performance metrics of OrgNet${}^{+}$ and other structure-based methods on the original (non-augmented) S669 dataset.^a^^,^^b^

		Direct			Reverse		
Method	Train	*r*	RMSE	MAE	*r*	RMSE	MAE
**OrgNet** ${}^{+}$	S2648-ensemble\S2648-original^b^	0.44 (0.58)	1.50 (1.08)	1.10 (0.84)	0.43 (0.57)	1.52 (1.12)	1.10 (0.84)
OrgNet (Buyanov *et al.* 2025)	S2648^c^	0.42 (0.54)	1.55 (1.16)	1.12 (0.86)	0.42 (0.55)	1.56 (1.17)	1.14 (0.88)
RaSP (Blaabjerg *et al.* 2023)		0.40 (0.55)	1.62 (1.16)	1.13 (0.83)	0.33 (0.35)	1.93 (1.68)	1.48 (1.30)
ThermoNet (Li *et al.* 2020)	Q1744	0.39 (0.55)	1.62 (1.24)	1.17 (0.93)	0.38 (0.51)	1.66 (1.32)	1.24 (1.02)
SDM (Worth *et al.* 2011)	S2648^d^	0.41 (0.56)	1.67 (1.33)	1.26 (1.02)	0.14 (0.12)	2.16 (1.98)	1.64 (1.52)
mCSM (Pires *et al.* 2014b)	S2648, S1925	0.36 (0.53)	1.54 (1.07)	1.13 (0.81)	0.22 (0.18)	2.30 (2.17)	1.86 (1.82)
DUET (Pires *et al.* 2014a)	S2648	0.41 (0.59)	1.52 (1.06)	1.10 (0.78)	0.23 (0.18)	2.14 (1.98)	1.68 (1.60)
Dynamut (Rodrigues *et al.* 2018)	S2648	0.42 (0.50)	1.60 (1.27)	1.19 (0.96)	0.34 (0.44)	1.70 (1.37)	1.24 (1.02)
ACDC-NN (Benevenuta *et al.* 2021)	S2648 + Varibench	**0.46** (0.61)	1.49 (1.07)	**1.05** (0.78)	**0.45** (0.60)	1.51 (1.08)	1.06 (0.78)
PremPS (Chen *et al.* 2020)	S2648	0.41 (**0.63**)	1.51 (1.03)	1.09 (0.80)	0.42 (**0.63**)	**1.49** (**0.99**)	**1.05** (**0.74**)
DDGun3D (Montanucci *et al.* 2019)	S2648, Varibench^d^	0.43 (**0.63**)	1.60 (1.11)	1.11 (0.81)	0.41 (0.58)	1.62 (1.18)	1.14 (0.86)
PoPMuSiC 2.1 (Dehouck *et al.* 2011)	S2648	0.42 (0.61)	1.51 (**1.02**)	1.09 (**0.76**)	0.24 (0.29)	2.09 (1.89)	1.64 (1.52)
INPS3D (Savojardo *et al.* 2016)	S2648	0.43 (0.61)	1.50 (**1.02**)	1.07 (**0.76**)	0.33 (0.35)	1.77 (1.48)	1.31 (1.12)
MAESTRO (Laimer *et al.* 2016)	S2648	**0.50** (**0.63**)	**1.44** (1.04)	**1.06** (0.79)	0.20 (0.20)	2.10 (1.93)	1.65 (1.54)
I-Mutant3.0 (Capriotti *et al.* 2005)	S2648^e^	0.36 (0.49)	1.54 (1.13)	1.12 (0.84)	0.15 (0.14)	2.32 (2.19)	1.87 (1.83)
FoldX (Schymkowitz *et al.* 2005)	–	0.21 (0.23)	2.32 (2.23)	1.57 (1.38)	0.22 (0.40)	2.48 (1.75)	1.50 (1.15)

a Values are reported for S669, and values in parentheses correspond to S461. Predictions for other models are sourced from Iqbal *et al.* (2022).

b Training set does not include experimental structures from S2648.

c 2333 protein structures for representation learning; ΔΔG from saturation mutagenesis of 55 proteins for stability training (Blaabjerg *et al.* 2023).

d Used as the benchmark.

e 2087 distinct single mutations from ProTherm (Bava *et al.* 2004).

Best values for each performance metric are highlighted in bold.

Finally, we evaluated OrgNet${}^{+}$ in a three-state classification setting, distinguishing destabilizing, neutral, and stabilizing mutations using experimentally motivated and validation-based thresholds. Specifically, we considered ±1.0 kcal/mol as defined for the S461 direct benchmark (Hernández *et al.* 2023) and ±0.5 kcal/mol used for the S669 direct benchmark (Pancotti *et al.* 2022). Because OrgNet${}^{+}$ is trained as a regression model and is not exposed to experimental ΔΔG decision thresholds during training, applying experimental cut-offs to predicted values, in general, is not optimal for classification. Accordingly, we additionally report results obtained using prediction thresholds selected by maximizing the mean Macro *F*1 score across cross-validation folds, yielding optimized prediction thresholds of ±0.75 and ±0.45 kcal/mol for the experimental classification thresholds of ±1.0 and ±0.5 kcal/mol, respectively (see Fig. 1, available as supplementary data at *Bioinformatics* online). On the augmented test sets, OrgNet${}^{+}$ achieved three-state classification performance that was comparable to or exceeded that of OrgNet and RaSP across all ensemble types (Tables 6–9, available as supplementary data at *Bioinformatics* online). Specifically, on the augmented S669 test set, OrgNet${}^{+}$ with optimized prediction thresholds attained Macro *F*1 scores of 0.44–0.48 and 0.43–0.46 across ensemble types for experimental thresholds of ±1.0 and ±0.5, respectively. A similar trend was observed for the augmented S461 test set (see Tables 6 and 7, available as supplementary data at *Bioinformatics* online). Ensemble-based training did not degrade classification performance on the original, non-augmented benchmarks. As shown in Tables 10–13, available as supplementary data at *Bioinformatics* online, OrgNet${}^{+}$ achieved three-state classification accuracy and Macro *F*1 scores on the original S461 and S669 datasets that were comparable to OrgNet, RaSP, and other state-of-the-art structure-based predictors. For example, on S461 with ±1.0 kcal/mol thresholds, OrgNet${}^{+}$ reached a Macro *F*1 of 0.52, comparable to PremPS (0.52) and RaSP (0.50). On S669, OrgNet${}^{+}$ achieved Macro *F*1 scores of 0.44, consistent with competing methods.

### 3.4 Ablation studies

To disentangle the contribution of individual conformational ensemble generators, we performed ablation studies in which OrgNet${}^{+}$ models were trained using a single-ensemble type and evaluated across all ensembles. These experiments revealed a clear tendency towards partial specialization: models trained on a given ensemble performed best when evaluated on the same ensemble type, while exhibiting degraded accuracy and increased uncertainty on unseen ensembles relative to the fully augmented OrgNet${}^{+}$ model trained on all five ensemble types (see Tables 4 and 5, available as supplementary data at *Bioinformatics* online). For example, on S669, OrgNet${}^{+}$ trained exclusively on Boltz-2 conformations achieved low uncertainty when tested on Boltz-2 ensembles ($\sigma_{\text{pred}}=0.27\pm 0.14$) but substantially higher uncertainty on NOLB ensembles ($\sigma_{\text{pred}}=0.66\pm 0.18$). A similar pattern was observed across other single-ensemble training regimes, particularly for structurally diverse ensembles such as NOLB and Backrub, which are characterized by lower average structural similarity. We further observed higher intra-ensemble similarity compared to inter-ensemble similarity, indicating that each conformational generator may have a specific sampling pattern with limited overlap across the generators (see Table 3, available as supplementary data at *Bioinformatics* online). These results indicate that naive data augmentation using a single conformational generator does not generalize well across different types of structural deformations.

As expected, training on the full combination of five ensemble types consistently yielded the most robust performance across all test conditions. On the full collection of augmented test sets, OrgNet${}^{+}$ achieved the lowest average prediction error and uncertainty on both S669 and S461 (Table 1), reducing prediction variance by 25%–30% relative to OrgNet. On the one hand, this demonstrates that exposure to diverse structural perturbations during training is critical for developing more robust models. On the other hand, this indicates that data augmentation is likely not the universal approach, as the resulting models may perform worse for protein structures obtained with a different modelling method.

We further examined the relationship between structural variability and prediction uncertainty by correlating ensemble-averaged $\bar{3D-\text{SSIM}}$with the prediction uncertainty ($\sigma_{\text{pred}}$). Across datasets, ensemble types, and models, we observed a consistent negative correlation between structural similarity and prediction uncertainty (see Table 1; Tables 4 and S5, available as supplementary data at *Bioinformatics* online), with correlation coefficients ranging from approximately −0.3 to −0.8. Notably, OrgNet${}^{+}$ exhibited the strongest correlations (e.g. r=−0.41 on S669 and r=−0.42 on S461 for the full collection of augmented datasets), indicating that residual prediction uncertainty closely tracks physically meaningful conformational variability rather than model instability. Together, these findings highlight both the benefits and limitations of data augmentation and motivate the development of principled approaches that explicitly account for structural uncertainty rather than relying solely on generator-specific perturbations.

## 4 Discussion

Proteins populate heterogeneous conformational landscapes rather than existing as single static structures, yet most structure-based stability predictors operate on a fixed reference conformation. Our results demonstrate that this mismatch constitutes a major and previously underappreciated source of uncertainty in structure-based ΔΔG prediction, specifically 3D CNN-based models. Even models that explicitly resolve SE(3) variance remain sensitive to flexible deformations, leading to large intra-ensemble prediction variability and, in some cases, contradictory stability assignments for the same point mutation. Thus, robustness to protein flexibility cannot be achieved by *post hoc* averaging predictions across multiple conformations; instead it should be an integral part of the optimization problem. The introduced OrgNet${}^{+}$ approach explicitly integrates proteins structure flexibility into the training, resulting in markedly reduced prediction variance and improved accuracy across diverse conformational ensemble types. Despite being trained exclusively on conformational ensembles and without access to single reference structures, OrgNet${}^{+}$ remains competitive on the original S461 and S669 benchmarks. This demonstrates that learning robustness to flexibility does not compromise performance on standard evaluation protocols and instead yields models that generalize more reliably across both reference and structurally heterogeneous inputs. At the same time, our ablation studies reveal important limitations of naive data augmentation approach: models trained on conformations generated by a single modelling method tend to specialize in the corresponding deformation patterns and exhibit degraded performance when evaluated on ensembles produced by different modelling methods. This behaviour highlights that data augmentation alone is not a universal solution: robustness learned to one type of structural perturbation does not necessarily transfer to others, suggesting that future progress will require approaches that go beyond conformational ensemble-based augmentation. All conformations in this study were treated as equally informative, whereas incorporating thermodynamic weighting or confidence-based schemes may further improve performance. Moreover, generated conformations capture only local perturbations around the reference structures, thus, more complex conformational transitions are not taken into account. Integrating methods for constructing compact and representative conformational ensembles—designed to summarize complex landscapes with a limited number of informative structures—may further enhance both robustness and interpretability (Conev *et al.* 2023). Rather than relying solely on increasingly diverse perturbation sets, more principled strategies that explicitly model structural uncertainty, deformation modes, or confidence-aware aggregation may offer a better generalization strategy. Finally, OrgNet${}^{+}$ is currently limited to the analysis of single-point mutations. Although it could, in principle, be used for multiple amino acid substitutions (e.g. via sequential application), the feasibility of such an approach requires rigorous evaluation on appropriate benchmarks. Moreover, this strategy is unlikely to capture compensatory or synergistic mutational effects. Incorporating protein dynamics and jointly modelling multiple substitution sites may help to address these limitations; however, this would require higher-order representations of amino acid substitutions and corresponding modifications to the neural network architecture.

## 5 Conclusion

In this work, we demonstrate that protein conformational flexibility constitutes a major and previously underappreciated source of uncertainty for structure-based ΔΔG prediction, even for modern 3D CNN models that explicitly resolve rigid-body invariances. By systematically analysing predictions across diverse conformational ensembles, we show that widely used predictors can yield highly variable—and sometimes contradictory—stability estimates for the same mutation. We address this limitation by introducing OrgNet${}^{+}$, an SE(3)-invariant 3D CNN approach that exposes the model to controlled structural variability during training. OrgNet${}^{+}$ consistently produces more robust and reliable predictions while simultaneously improving accuracy across multiple benchmarks and various conformational ensemble generator methods, and remains competitive on standard single reference structure evaluations. Our ablation studies further reveal that robustness cannot be achieved through naive or conformational generator-specific data augmentation alone, highlighting the need for principled approaches accounting for protein structural flexibility.

## Supplementary Material

btag258_Supplementary_Data

## Data Availability

The OrgNet${}^{+}$ model, weights, and code for orientation standardization, voxelization, and ΔΔG predictions are publicly available at https://github.com/i-Molecule/OrgNet. RaSP model and scripts were obtained from https://github.com/KULL-Centre/_2022_ML-ddG-Blaabjerg/. Predictions for other structure-based methods were taken from (Iqbal *et al.* 2022). Predictions for both the original and conformationally augmented S669 and S461 benchmarks are publicly available on Zenodo at https://doi.org/10.5281/zenodo.19211983.
