# Supplementary material for: OrgNet+: towards robust protein stability prediction with convolutional neural networks
Source: Bioinformatics. 2026 Jul 7;42(Suppl 1):btag258. doi: 10.1093/bioinformatics/btag258 (PMC13340226; doi:10.1093/bioinformatics/btag258)
Supplement: btag258_Supplementary_Data [file btag258_supplementary_data.pdf]

Bioinformatics, 2026, pp. 1–1

doi:
ISMB 2026, Conference Proceedings

ISMB 2026, CONFERENCE PROCEEDINGS

Supplementary information for: OrgNet^{+}: towards robust protein stability prediction with convolutional neural networks

Anastasia Sarycheva,[1,2] Aleksandr Shumilov[1,2] and Petr Popov[1,2,*]

[1]Constructor Knowledge Labs, Bremen, 28759, Germany and [2]Constructor University Bremen gGmbH, Bremen, 28759, Germany
[*]Corresponding author. ppopov@constructor.university

Abstract

Supplementary information for OrgNet^{+}: towards robust protein stability prediction with convolutional neural networks.

Three-state classification

For OrgNet^{+}, the predicted $\Delta\Delta G$ threshold used for three-state classification was selected by maximizing the Macro F1 score across five-fold cross-validation. For each cross-validation split, predictions on the validation set were evaluated over a range of candidate predicted $\Delta\Delta G$ thresholds. At each threshold, predictions were mapped to three classes (destabilizing, neutral, stabilizing) according to fixed experimental $\Delta\Delta G$ cutoffs, and the corresponding Macro F1 score was computed. The final threshold was chosen as the value that maximized the mean Macro F1 score averaged over the five validation folds.

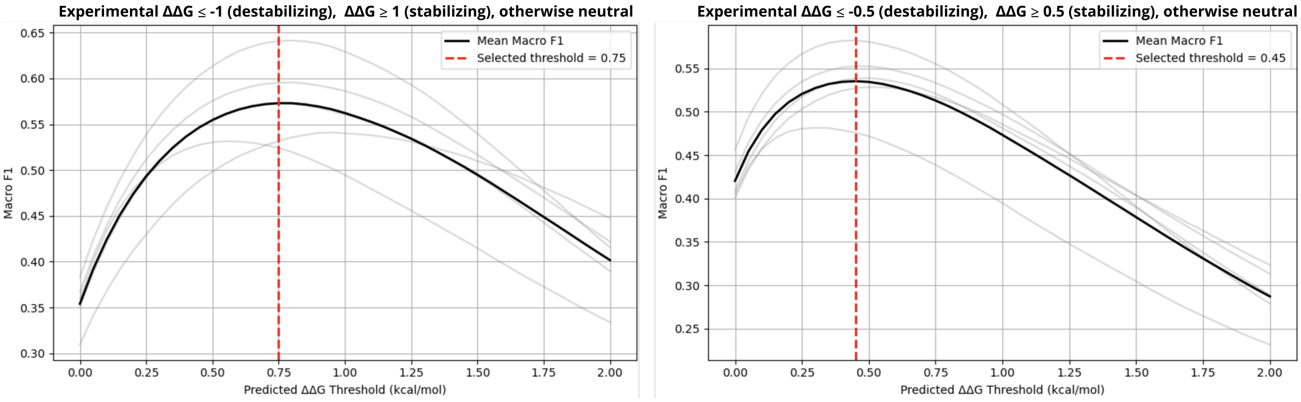

Fig. S1. Selection of the predicted $\Delta\Delta G$ threshold for three-state classification with OrgNet^{+}. Macro F1 score is shown as a function of the predicted $\Delta\Delta G$ threshold for two experimental class definitions: (left) destabilizing $\Delta\Delta G \leq -1$, stabilizing $\Delta\Delta G \geq 1$, neutral otherwise; (right) destabilizing $\Delta\Delta G \leq -0.5$, stabilizing $\Delta\Delta G \geq 0.5$, neutral otherwise. Grey curves correspond to Macro F1 scores computed on individual validation folds of five-fold cross-validation, while the black curve indicates the mean Macro F1 across folds. The red dashed line marks the selected predicted threshold that maximizes the mean Macro F1 score across validation folds.

© The Author 2026. Published by Oxford University Press. All rights reserved. For permissions, please e-mail: journals.permissions@oup.com

Table S1. Wall-clock runtime for generating 100-conformation ensembles per protein (evaluated on the S2648 dataset) using different methods: NOLB (`https://team.inria.fr/nano-d/software/nolb-normal-modes/`); iMod (`https://chaconlab.org/multiscale-simulations/imod`); Backrub (`https://www.rosettacommons.org/docs/latest/application_documentation/backrub`); Boltz-2 (`https://github.com/jwohlwend/boltz`; `https://colabfold.mmseqs.com/`); MD (`https://www.gromacs.org/`). Reported are total runtime for the full dataset, as well as average runtime per protein in seconds, together with the corresponding compute configuration.

Method	Whole dataset (hours)	Mean ± SD (seconds/protein)	Compute profile
NOLB	0.12	3.16 ± 2.09	Intel(R) Xeon(R) w7-3455 processor (24 cores)
iMod	0.01	0.28 ± 0.19	Intel(R) Xeon(R) w7-3455 processor (24 cores)
Backrub	24.89	678.68 ± 567.82	Intel(R) Xeon(R) w7-3455 processor (24 cores)
Boltz-2	5.04	137.34 ± 69.42	1x NVIDIA GeForce RTX 4090
MD	38.66	1070.61 ± 387.21	1x NVIDIA GeForce RTX 4090 + 24 CPU cores

Table S2. Key parameters used in molecular dynamics (MD) simulation.

Parameter	Value	Description
`simulation_time`	10000	Total production simulation time in picoseconds (ps).
`simulation_dt`	0.002	Integration time step in picoseconds (ps), i.e., 2 fs.
`nvt_equilibration_time`	1000	Duration of the NVT (constant volume and temperature) equilibration phase in ps.
`npt_equilibration_time`	1000	Duration of the NPT (constant pressure and temperature) equilibration phase in ps.
`openmm_ionic_strength`	0.15	Ionic strength (mol/L) of the solvent.
`box_protein_padding`	2.0	Padding between protein surface and box edge in nm.
`forcefield`	`amber14sb`	Biomolecular force field used for protein atoms.
`gro_water_model`	`tip3p`	Water model used for solvation.
`thermostat`	`v-rescale`	Temperature coupling algorithm used for equilibration and production.
`thermostat_ref_t`	300	Target temperature (K).
`thermostat_tau_t_eq`	0.1	Thermostat coupling time constant (ps) during NVT/NPT equilibration.
`thermostat_tau_t_prod`	0.5	Thermostat coupling time constant (ps) during production MD.
`barostat`	`C-rescale`	Pressure coupling algorithm used during NPT equilibration and production.
`barostat_ref_p`	1.0	Target pressure (bar).
`barostat_pcoupltype`	`isotropic`	Pressure coupling scheme.
`barostat_tau_p_eq`	2.0	Barostat coupling time constant (ps) during NPT equilibration.
`barostat_tau_p_prod`	5.0	Barostat coupling time constant (ps) during production MD.

Table S3. Pairwise structural similarity between conformational ensemble generation methods, measured as mean ± standard deviation (SD) of $\overline{\text{3D-SSIM}}$ scores across point-mutations. For each dataset (S669 and S461, top; S2648, bottom), matrices report all-to-all comparisons between conformations generated by different methods. Diagonal entries (**bold**) correspond to within-ensemble similarity (ensemble-averaged $\overline{\text{3D-SSIM}}$), while off-diagonal entries represent cross-method comparisons. The "Full" row reports the mean across all five ensemble methods based on within-ensemble statistics.

S669

	Backrub	Boltz-2	iMod	MD	NOLB
Backrub	**0.54 ± 0.12**	0.44 ± 0.15	0.46 ± 0.15	0.43 ± 0.14	0.36 ± 0.11
Boltz-2	0.44 ± 0.15	**0.87 ± 0.13**	0.64 ± 0.19	0.66 ± 0.16	0.45 ± 0.17
iMod	0.46 ± 0.15	0.64 ± 0.19	**0.73 ± 0.19**	0.57 ± 0.17	0.48 ± 0.20
MD	0.43 ± 0.14	0.66 ± 0.16	0.57 ± 0.17	**0.67 ± 0.11**	0.41 ± 0.15
NOLB	0.36 ± 0.11	0.45 ± 0.17	0.48 ± 0.20	0.41 ± 0.15	**0.42 ± 0.19**
Full			0.64 ± 0.09		

S461

	Backrub	Boltz-2	iMod	MD	NOLB
Backrub	**0.53 ± 0.12**	0.43 ± 0.15	0.44 ± 0.16	0.42 ± 0.15	0.35 ± 0.11
Boltz-2	0.43 ± 0.15	**0.87 ± 0.13**	0.64 ± 0.19	0.67 ± 0.16	0.45 ± 0.18
iMod	0.44 ± 0.16	0.64 ± 0.19	**0.75 ± 0.20**	0.58 ± 0.17	0.50 ± 0.21
MD	0.42 ± 0.15	0.67 ± 0.16	0.58 ± 0.17	**0.68 ± 0.11**	0.42 ± 0.16
NOLB	0.35 ± 0.11	0.45 ± 0.18	0.50 ± 0.21	0.42 ± 0.16	**0.44 ± 0.20**
Full			0.65 ± 0.09		

S2648

	Backrub	Boltz-2	iMod	MD	NOLB
Backrub	**0.53 ± 0.10**	0.47 ± 0.13	0.44 ± 0.11	0.44 ± 0.12	0.33 ± 0.10
Boltz-2	0.47 ± 0.13	**0.91 ± 0.08**	0.62 ± 0.15	0.70 ± 0.14	0.42 ± 0.15
iMod	0.44 ± 0.11	0.62 ± 0.15	**0.61 ± 0.15**	0.56 ± 0.13	0.39 ± 0.14
MD	0.44 ± 0.12	0.70 ± 0.14	0.56 ± 0.13	**0.70 ± 0.09**	0.39 ± 0.13
NOLB	0.33 ± 0.10	0.42 ± 0.15	0.39 ± 0.14	0.39 ± 0.13	**0.35 ± 0.15**
Full			0.62 ± 0.07		

Table S4. Performance metrics in direct and reverse settings for full augmented test datasets (i.e. all 5 conformational ensembles) as well as for each conformational ensemble separately (Backrub, Boltz-2, iMod, MD, NOLB) – S669. Reported are prediction accuracy (RMSE, MAE, Pearson r), prediction uncertainty (σ_{pred}) introduced by conformational flexibility, and the correlation between prediction uncertainty (σ_{pred}) and conformational flexibility expressed by $\overline{\text{3D-SSIM}}$.

**Method**	**Test**	Direct					Reverse				
		**RMSE**	**MAE**	**r**	σ_{pred}	$r_{\overline{\text{3D-SSIM}}\text{-}\sigma_{\text{pred}}}$	**RMSE**	**MAE**	**r**	σ_{pred}	$r_{\overline{\text{3D-SSIM}}\text{-}\sigma_{\text{pred}}}$
RaSP	full	1.60	1.13	0.37	0.474 ± 0.278	-0.29	1.97	1.50	0.26	0.400 ± 0.209	-0.30
	Backrub	1.63	1.18	0.31	0.372 ± 0.207	-0.23	1.92	1.46	0.25	0.313 ± 0.159	-0.16
	Boltz-2	1.52	1.05	0.46	0.216 ± 0.160	-0.32	1.92	1.45	0.30	0.202 ± 0.139	-0.34
	iMod	1.60	1.14	0.37	0.293 ± 0.256	-0.68	1.99	1.52	0.27	0.305 ± 0.221	-0.61
	MD	1.59	1.11	0.42	0.299 ± 0.167	-0.36	1.93	1.47	0.31	0.286 ± 0.159	-0.36
	NOLB	1.64	1.18	0.32	0.457 ± 0.251	-0.47	2.09	1.60	0.21	0.466 ± 0.285	-0.41
OrgNet	full	1.66	1.22	0.31	0.570 ± 0.225	-0.29	1.68	1.24	0.31	0.593 ± 0.237	-0.27
	Backrub	1.80	1.36	0.19	0.449 ± 0.185	-0.19	1.81	1.37	0.20	0.464 ± 0.200	-0.13
	Boltz-2	1.50	1.09	0.41	0.310 ± 0.163	-0.34	1.54	1.11	0.40	0.315 ± 0.166	-0.33
	iMod	1.64	1.20	0.33	0.444 ± 0.215	-0.70	1.68	1.24	0.31	0.465 ± 0.225	-0.67
	MD	1.58	1.15	0.38	0.445 ± 0.176	-0.41	1.61	1.17	0.37	0.464 ± 0.184	-0.43
	NOLB	1.74	1.30	0.25	0.620 ± 0.183	-0.38	1.76	1.32	0.25	0.642 ± 0.192	-0.37
OrgNet^{+} (full)	full	**1.54**	**1.13**	**0.41**	0.420 ± 0.130	-0.41	**1.56**	**1.14**	**0.40**	0.430 ± 0.134	-0.39
	Backrub	1.60	1.19	0.36	0.383 ± 0.119	-0.48	1.64	1.22	0.34	0.398 ± 0.127	-0.45
	Boltz-2	1.43	1.02	0.46	0.199 ± 0.103	-0.59	1.46	1.04	0.45	0.209 ± 0.112	-0.56
	iMod	1.53	1.12	0.42	0.308 ± 0.155	-0.80	1.56	1.13	0.41	0.316 ± 0.161	-0.78
	MD	1.51	1.11	0.44	0.306 ± 0.103	-0.56	1.53	1.11	0.43	0.314 ± 0.109	-0.54
	NOLB	1.58	1.17	0.37	0.483 ± 0.145	-0.69	1.61	1.18	0.36	0.488 ± 0.142	-0.67
OrgNet^{+} (Backrub)	full	1.58	1.17	0.38	0.373 ± 0.116	-0.41	1.58	1.16	0.37	0.372 ± 0.113	-0.41
	Backrub	1.59	1.19	0.37	0.351 ± 0.105	-0.48	1.62	1.19	0.34	0.359 ± 0.108	-0.47
	Boltz-2	1.50	1.10	0.43	0.174 ± 0.093	-0.64	1.48	1.07	0.42	0.179 ± 0.090	-0.62
	iMod	1.61	1.19	0.37	0.272 ± 0.135	-0.77	1.59	1.16	0.38	0.273 ± 0.138	-0.76
	MD	1.55	1.14	0.41	0.267 ± 0.085	-0.53	1.55	1.13	0.40	0.270 ± 0.086	-0.54
	NOLB	1.66	1.24	0.34	0.437 ± 0.134	-0.67	1.65	1.22	0.33	0.432 ± 0.131	-0.69
OrgNet^{+} (Boltz-2)	Backrub	1.61	1.20	0.38	0.555 ± 0.131	-0.39	1.58	1.16	0.37	0.533 ± 0.122	-0.40
	Backrub	1.74	1.33	0.32	0.511 ± 0.125	-0.43	1.65	1.23	0.30	0.514 ± 0.126	-0.41
	Boltz-2	1.45	1.06	0.46	0.267 ± 0.136	-0.63	1.46	1.05	0.44	0.268 ± 0.133	-0.63
	iMod	1.59	1.19	0.38	0.398 ± 0.176	-0.80	1.56	1.14	0.39	0.397 ± 0.176	-0.76
	MD	1.55	1.15	0.42	0.406 ± 0.118	-0.47	1.55	1.13	0.40	0.397 ± 0.113	-0.51
	NOLB	1.68	1.27	0.31	0.658 ± 0.176	-0.75	1.67	1.24	0.31	0.655 ± 0.196	-0.73
OrgNet^{+} (iMod)	full	1.56	1.15	0.40	0.448 ± 0.119	-0.41	1.56	1.14	0.39	0.455 ± 0.123	-0.39
	Backrub	1.64	1.23	0.35	0.415 ± 0.123	-0.47	1.65	1.22	0.33	0.428 ± 0.132	-0.45
	Boltz-2	1.46	1.04	0.45	0.207 ± 0.108	-0.65	1.45	1.03	0.46	0.214 ± 0.114	-0.65
	iMod	1.54	1.13	0.41	0.322 ± 0.154	-0.81	1.55	1.12	0.40	0.329 ± 0.157	-0.79
	MD	1.54	1.13	0.43	0.316 ± 0.098	-0.55	1.53	1.11	0.42	0.322 ± 0.102	-0.54
	NOLB	1.61	1.20	0.36	0.540 ± 0.160	-0.77	1.61	1.19	0.35	0.542 ± 0.155	-0.75
OrgNet^{+} (MD)	full	1.57	1.16	0.39	0.488 ± 0.145	-0.33	1.58	1.15	0.38	0.477 ± 0.140	-0.34
	Backrub	1.68	1.26	0.33	0.449 ± 0.137	-0.43	1.65	1.23	0.31	0.457 ± 0.141	-0.42
	Boltz-2	1.44	1.03	0.46	0.223 ± 0.127	-0.60	1.46	1.04	0.45	0.225 ± 0.122	-0.62
	iMod	1.56	1.15	0.40	0.339 ± 0.166	-0.77	1.56	1.14	0.39	0.341 ± 0.171	-0.75
	MD	1.52	1.12	0.43	0.341 ± 0.114	-0.47	1.54	1.11	0.41	0.338 ± 0.113	-0.54
	NOLB	1.64	1.22	0.33	0.571 ± 0.181	-0.71	1.66	1.23	0.32	0.569 ± 0.185	-0.72
OrgNet^{+} (NOLB)	full	1.56	1.14	0.40	0.380 ± 0.131	-0.41	1.56	1.14	0.40	0.381 ± 0.129	-0.43
	Backrub	1.64	1.21	0.35	0.342 ± 0.126	-0.46	1.64	1.22	0.34	0.347 ± 0.127	-0.47
	Boltz-2	1.48	1.06	0.44	0.173 ± 0.095	-0.48	1.47	1.05	0.45	0.175 ± 0.094	-0.51
	iMod	1.55	1.13	0.41	0.280 ± 0.152	-0.81	1.55	1.13	0.40	0.279 ± 0.150	-0.78
	MD	1.55	1.13	0.42	0.264 ± 0.096	-0.52	1.54	1.12	0.42	0.266 ± 0.098	-0.56
	NOLB	1.58	1.16	0.37	0.432 ± 0.141	-0.64	1.58	1.16	0.37	0.435 ± 0.140	-0.65

Table S5. Performance metrics in direct and reverse settings for full augmented test datasets (i.e. all 5 conformational ensembles) as well as for each conformational ensemble separately (Backrub, Boltz-2, iMod, MD, NOLB) – S461. Reported are prediction accuracy (RMSE, MAE, Pearson r), prediction uncertainty (σ_{pred}) introduced by conformational flexibility, and the correlation between prediction uncertainty (σ_{pred}) and conformational flexibility expressed by $\overline{\text{3D-SSIM}}$.

**Method**	**Test**	Direct					Reverse				
		**RMSE**	**MAE**	**r**	$\sigma_{\textbf{pred}}$	$r_{\overline{\text{3D-SSIM}}\text{-}\sigma_{\text{pred}}}$	**RMSE**	**MAE**	**r**	$\sigma_{\textbf{pred}}$	$r_{\overline{\text{3D-SSIM}}\text{-}\sigma_{\text{pred}}}$
RaSP	full	1.17	0.84	0.52	0.474 ± 0.283	-0.31	1.73	1.34	0.30	0.381 ± 0.199	-0.33
	Backrub	1.24	0.91	0.43	0.380 ± 0.206	-0.29	1.65	1.28	0.28	0.298 ± 0.154	-0.24
	Boltz-2	1.16	0.80	0.60	0.214 ± 0.167	-0.32	1.69	1.30	0.35	0.197 ± 0.128	-0.36
	iMod	1.14	0.83	0.53	0.258 ± 0.246	-0.77	1.75	1.35	0.28	0.275 ± 0.214	-0.66
	MD	1.11	0.78	0.59	0.285 ± 0.154	-0.38	1.67	1.30	0.34	0.274 ± 0.151	-0.37
	NOLB	1.19	0.88	0.47	0.439 ± 0.243	-0.49	1.86	1.45	0.24	0.444 ± 0.275	-0.47
OrgNet	full	1.32	0.99	0.43	0.576 ± 0.245	-0.32	1.34	1.01	0.44	0.595 ± 0.256	-0.27
	Backrub	1.54	1.19	0.28	0.449 ± 0.197	-0.28	1.54	1.20	0.30	0.460 ± 0.216	-0.25
	Boltz-2	1.16	0.86	0.54	0.301 ± 0.160	-0.33	1.16	0.87	0.56	0.305 ± 0.158	-0.34
	iMod	1.27	0.95	0.47	0.419 ± 0.227	-0.75	1.32	0.99	0.45	0.436 ± 0.235	-0.70
	MD	1.18	0.89	0.53	0.427 ± 0.178	-0.45	1.19	0.89	0.54	0.441 ± 0.178	-0.46
	NOLB	1.41	1.08	0.37	0.614 ± 0.192	-0.40	1.44	1.10	0.37	0.627 ± 0.194	-0.37
OrgNet$^+$ (full)	full	**1.15**	**0.88**	**0.54**	0.418 ± 0.137	-0.42	**1.18**	**0.89**	**0.53**	0.426 ± 0.140	-0.37
	Backrub	1.24	0.95	0.47	0.389 ± 0.124	-0.48	1.29	1.00	0.46	0.406 ± 0.134	-0.44
	Boltz-2	1.07	0.81	0.60	0.196 ± 0.104	-0.60	1.10	0.82	0.60	0.206 ± 0.112	-0.57
	iMod	1.13	0.86	0.55	0.286 ± 0.164	-0.84	1.16	0.87	0.55	0.294 ± 0.169	-0.81
	MD	1.09	0.83	0.58	0.295 ± 0.101	-0.59	1.12	0.83	0.58	0.299 ± 0.106	-0.57
	NOLB	1.21	0.92	0.50	0.476 ± 0.153	-0.70	1.24	0.94	0.49	0.479 ± 0.147	-0.67
OrgNet$^+$ (Backrub)	full	1.23	0.95	0.49	0.376 ± 0.119	-0.46	1.21	0.92	0.49	0.373 ± 0.115	-0.42
	Backrub	1.23	0.95	0.48	0.360 ± 0.104	-0.46	1.25	0.95	0.46	0.369 ± 0.110	-0.44
	Boltz-2	1.17	0.90	0.54	0.171 ± 0.092	-0.63	1.14	0.87	0.54	0.176 ± 0.087	-0.64
	iMod	1.26	0.97	0.47	0.258 ± 0.147	-0.82	1.22	0.91	0.49	0.257 ± 0.147	-0.81
	MD	1.17	0.89	0.54	0.260 ± 0.086	-0.53	1.15	0.87	0.53	0.259 ± 0.084	-0.58
	NOLB	1.33	1.03	0.44	0.439 ± 0.141	-0.71	1.30	0.98	0.44	0.430 ± 0.136	-0.72
OrgNet$^+$ (Boltz-2)	full	1.28	0.98	0.48	0.555 ± 0.135	-0.39	1.21	0.92	0.49	0.530 ± 0.122	-0.42
	Backrub	1.46	1.14	0.41	0.521 ± 0.123	-0.35	1.30	1.00	0.39	0.525 ± 0.128	-0.35
	Boltz-2	1.13	0.86	0.57	0.268 ± 0.141	-0.62	1.13	0.85	0.56	0.264 ± 0.132	-0.64
	iMod	1.23	0.95	0.50	0.368 ± 0.182	-0.85	1.17	0.88	0.52	0.371 ± 0.183	-0.81
	MD	1.17	0.90	0.55	0.396 ± 0.119	-0.45	1.14	0.86	0.54	0.382 ± 0.111	-0.53
	NOLB	1.36	1.05	0.41	0.653 ± 0.183	-0.76	1.31	0.99	0.43	0.646 ± 0.198	-0.79
OrgNet$^+$ (iMod)	full	1.20	0.91	0.53	0.447 ± 0.122	-0.42	1.17	0.88	0.53	0.454 ± 0.125	-0.39
	Backrub	1.30	1.00	0.46	0.425 ± 0.125	-0.42	1.28	0.99	0.45	0.442 ± 0.136	-0.40
	Boltz-2	1.11	0.83	0.58	0.205 ± 0.110	-0.66	1.09	0.81	0.60	0.212 ± 0.114	-0.66
	iMod	1.16	0.88	0.54	0.298 ± 0.162	-0.86	1.13	0.85	0.55	0.305 ± 0.164	-0.82
	MD	1.14	0.86	0.57	0.306 ± 0.097	-0.58	1.11	0.82	0.58	0.309 ± 0.097	-0.55
	NOLB	1.25	0.96	0.48	0.536 ± 0.168	-0.79	1.23	0.93	0.48	0.536 ± 0.160	-0.77
OrgNet$^+$ (MD)	full	1.22	0.93	0.50	0.496 ± 0.153	-0.34	1.21	0.91	0.50	0.481 ± 0.142	-0.35
	Backrub	1.37	1.06	0.43	0.467 ± 0.141	-0.35	1.31	1.01	0.40	0.474 ± 0.145	-0.35
	Boltz-2	1.10	0.82	0.57	0.224 ± 0.130	-0.60	1.12	0.83	0.57	0.221 ± 0.121	-0.62
	iMod	1.19	0.90	0.51	0.317 ± 0.176	-0.83	1.17	0.87	0.53	0.321 ± 0.182	-0.80
	MD	1.12	0.85	0.57	0.335 ± 0.115	-0.46	1.13	0.84	0.56	0.325 ± 0.110	-0.55
	NOLB	1.30	1.00	0.44	0.573 ± 0.192	-0.73	1.30	0.99	0.45	0.566 ± 0.190	-0.76
OrgNet$^+$ (NOLB)	full	1.18	0.89	0.54	0.377 ± 0.139	-0.40	1.17	0.88	0.54	0.376 ± 0.135	-0.43
	Backrub	1.28	0.98	0.48	0.351 ± 0.136	-0.45	1.27	0.98	0.48	0.354 ± 0.136	-0.47
	Boltz-2	1.13	0.85	0.57	0.171 ± 0.097	-0.48	1.11	0.83	0.59	0.172 ± 0.094	-0.51
	iMod	1.15	0.86	0.55	0.259 ± 0.161	-0.83	1.14	0.86	0.56	0.258 ± 0.158	-0.81
	MD	1.14	0.86	0.57	0.255 ± 0.096	-0.56	1.13	0.84	0.58	0.253 ± 0.096	-0.59
	NOLB	1.19	0.90	0.51	0.422 ± 0.147	-0.65	1.19	0.90	0.51	0.425 ± 0.147	-0.64

Table S6. Three-state classification performance on S461 (direct). Destabilizing if $\Delta\Delta G \leq -1$ kcal/mol, stabilizing if $\Delta\Delta G \geq 1$ kcal/mol, otherwise neutral. Reported are strict accuracy (Sc), off-by-one (Of1), off-by-two (Of2), Macro F1, and class-wise F1 scores.

Method	Test	Sc	Of1	Of2	Macro F1	F1 Destab	F1 Neutral	F1 Stab
RaSP	full	0.70	0.29	0.01	0.48	0.69	0.73	0.02
	Backrub	0.65	0.34	0.00	0.43	0.57	0.72	0.01
	Boltz-2	0.73	0.26	0.01	0.50	0.74	0.74	0.02
	iMod	0.71	0.28	0.01	0.49	0.71	0.74	0.03
	MD	0.74	0.25	0.01	0.51	0.74	0.76	0.04
	NOLB	0.68	0.31	0.01	0.46	0.66	0.72	0.01
OrgNet	full	0.63	0.36	0.01	0.44	0.54	0.70	0.10
	Backrub	0.54	0.46	0.00	0.33	0.25	0.67	0.06
	Boltz-2	0.68	0.31	0.01	0.51	0.65	0.72	0.14
	iMod	0.65	0.35	0.01	0.47	0.57	0.71	0.13
	MD	0.68	0.32	0.01	0.48	0.63	0.73	0.09
	NOLB	0.60	0.39	0.01	0.41	0.49	0.68	0.05
OrgNet^+ (full)	full	0.67	0.32	0.01	0.51	0.64	0.72	0.17
	Backrub	0.64	0.35	0.01	0.48	0.56	0.70	0.18
	Boltz-2	0.70	0.29	0.00	0.55	0.69	0.73	0.22
	iMod	0.68	0.31	0.01	0.50	0.65	0.72	0.13
	MD	0.69	0.30	0.01	0.53	0.67	0.73	0.20
	NOLB	0.65	0.34	0.01	0.48	0.61	0.71	0.12
OrgNet^+ (full) *	full	0.66	0.33	0.01	0.51	0.67	0.68	0.19
	Backrub	0.64	0.35	0.01	0.50	0.62	0.68	0.19
	Boltz-2	0.68	0.31	0.01	0.54	0.70	0.68	0.23
	iMod	0.67	0.32	0.01	0.51	0.68	0.68	0.17
	MD	0.68	0.31	0.01	0.53	0.69	0.70	0.21
	NOLB	0.64	0.34	0.01	0.49	0.65	0.67	0.15

*Selected prediction threshold $\Delta\Delta G = 0.75$ based on validation three-state Macro F1.

Table S7. Three-state classification performance on S461 (direct). Destabilizing if $\Delta\Delta G \leq -0.5$ kcal/mol, stabilizing if $\Delta\Delta G \geq 0.5$ kcal/mol, otherwise neutral. Reported are strict accuracy (Sc), off-by-one (Of1), off-by-two (Of2), Macro F1, and class-wise F1 scores.

Method	Test	Sc	Of1	Of2	Macro F1	F1 Destab	F1 Neutral	F1 Stab
RaSP	full	0.66	0.31	0.03	0.46	0.76	0.52	0.10
	Backrub	0.62	0.36	0.03	0.44	0.71	0.52	0.08
	Boltz-2	0.69	0.28	0.03	0.48	0.79	0.53	0.12
	iMod	0.66	0.31	0.03	0.46	0.76	0.51	0.10
	MD	0.69	0.29	0.02	0.48	0.79	0.54	0.11
	NOLB	0.64	0.33	0.03	0.45	0.75	0.51	0.09
OrgNet	full	0.54	0.42	0.05	0.41	0.62	0.48	0.13
	Backrub	0.41	0.55	0.04	0.32	0.38	0.46	0.10
	Boltz-2	0.60	0.36	0.04	0.46	0.71	0.48	0.19
	iMod	0.57	0.39	0.04	0.43	0.66	0.49	0.14
	MD	0.60	0.36	0.04	0.45	0.70	0.51	0.14
	NOLB	0.51	0.42	0.07	0.39	0.60	0.46	0.11
OrgNet^+ (full)	full	0.61	0.35	0.04	0.47	0.72	0.48	0.21
	Backrub	0.57	0.38	0.05	0.45	0.68	0.48	0.18
	Boltz-2	0.63	0.33	0.03	0.50	0.74	0.49	0.27
	iMod	0.62	0.35	0.03	0.46	0.72	0.48	0.19
	MD	0.63	0.34	0.04	0.49	0.73	0.50	0.24
	NOLB	0.59	0.36	0.04	0.45	0.70	0.47	0.17
OrgNet^+ (full)*	full	0.61	0.34	0.04	0.47	0.72	0.47	0.21
	Backrub	0.58	0.37	0.05	0.44	0.69	0.46	0.18
	Boltz-2	0.64	0.32	0.04	0.50	0.75	0.48	0.27
	iMod	0.62	0.34	0.04	0.47	0.73	0.47	0.20
	MD	0.63	0.33	0.04	0.49	0.74	0.49	0.24
	NOLB	0.59	0.36	0.05	0.44	0.71	0.46	0.17

*Selected prediction threshold $\Delta\Delta G = 0.45$ based on validation three-state Macro F1.

Table S8. Three-state classification performance on S669 (direct). Destabilizing if $\Delta\Delta G \leq -1$ kcal/mol, stabilizing if $\Delta\Delta G \geq 1$ kcal/mol, otherwise neutral. Reported are strict accuracy (Sc), off-by-one (Of1), off-by-two (Of2), Macro F1, and class-wise F1 scores.

Method	Test	Sc	Of1	Of2	Macro F1	F1 Destab	F1 Neutral	F1 Stab
RaSP	full	0.62	0.36	0.02	0.43	0.61	0.67	0.02
	Backrub	0.58	0.40	0.02	0.39	0.51	0.66	0.01
	Boltz-2	0.66	0.32	0.02	0.47	0.68	0.69	0.03
	iMod	0.61	0.37	0.02	0.43	0.61	0.66	0.01
	MD	0.64	0.34	0.02	0.45	0.66	0.67	0.02
	NOLB	0.59	0.38	0.03	0.41	0.58	0.65	0.01
OrgNet	full	0.56	0.42	0.02	0.40	0.47	0.65	0.09
	Backrub	0.50	0.49	0.01	0.32	0.24	0.64	0.07
	Boltz-2	0.61	0.38	0.01	0.45	0.58	0.67	0.11
	iMod	0.57	0.41	0.02	0.42	0.49	0.65	0.11
	MD	0.59	0.39	0.02	0.44	0.55	0.66	0.11
	NOLB	0.53	0.45	0.02	0.37	0.42	0.63	0.07
OrgNet^{+} (full)	full	0.59	0.39	0.02	0.44	0.56	0.66	0.10
	Backrub	0.56	0.42	0.02	0.42	0.49	0.65	0.11
	Boltz-2	0.63	0.36	0.01	0.46	0.63	0.68	0.09
	iMod	0.59	0.39	0.02	0.43	0.57	0.66	0.07
	MD	0.60	0.38	0.02	0.45	0.58	0.66	0.12
	NOLB	0.57	0.41	0.02	0.42	0.53	0.65	0.09
OrgNet^{+} (full)*	full	0.58	0.39	0.02	0.46	0.60	0.62	0.15
	Backrub	0.56	0.41	0.03	0.45	0.56	0.62	0.17
	Boltz-2	0.61	0.37	0.02	0.47	0.65	0.63	0.13
	iMod	0.58	0.39	0.02	0.45	0.61	0.62	0.13
	MD	0.59	0.38	0.02	0.48	0.62	0.62	0.19
	NOLB	0.57	0.40	0.03	0.44	0.58	0.61	0.14

*Selected prediction threshold $\Delta\Delta G = 0.75$ based on validation three-state Macro F1.

Table S9. Three-state classification performance on S669 (direct). Destabilizing if $\Delta\Delta G \leq -0.5$ kcal/mol, stabilizing if $\Delta\Delta G \geq 0.5$ kcal/mol, otherwise neutral. Reported are strict accuracy (Sc), off-by-one (Of1), off-by-two (Of2), Macro F1, and class-wise F1 scores.

Method	Test	Sc	Of1	Of2	Macro F1	F1 Destab	F1 Neutral	F1 Stab
RaSP	full	0.57	0.37	0.06	0.42	0.70	0.45	0.13
	backrub	0.53	0.41	0.06	0.40	0.65	0.45	0.11
	boltz	0.62	0.33	0.05	0.46	0.74	0.47	0.18
	imodv03	0.56	0.37	0.06	0.42	0.70	0.43	0.13
	md	0.59	0.35	0.06	0.44	0.72	0.45	0.14
	NOLB	0.55	0.38	0.07	0.41	0.68	0.44	0.10
OrgNet	full	0.48	0.45	0.07	0.40	0.57	0.43	0.21
	backrub	0.38	0.55	0.06	0.33	0.38	0.43	0.19
	boltz	0.53	0.41	0.06	0.44	0.64	0.43	0.25
	imodv03	0.49	0.43	0.07	0.42	0.60	0.43	0.22
	md	0.52	0.41	0.07	0.43	0.63	0.45	0.22
	NOLB	0.45	0.45	0.10	0.38	0.55	0.42	0.18
OrgNet^{+} (full)	full	0.54	0.40	0.06	0.44	0.67	0.42	0.24
	backrub	0.51	0.42	0.07	0.43	0.63	0.41	0.25
	boltz	0.57	0.38	0.05	0.46	0.70	0.43	0.24
	imodv03	0.54	0.40	0.06	0.44	0.67	0.41	0.22
	md	0.55	0.39	0.06	0.46	0.68	0.43	0.27
	NOLB	0.52	0.40	0.07	0.43	0.65	0.41	0.22
OrgNet^{+} (full)*	full	0.54	0.39	0.07	0.45	0.68	0.41	0.26
	backrub	0.51	0.41	0.08	0.43	0.64	0.40	0.27
	boltz	0.57	0.37	0.06	0.46	0.71	0.42	0.25
	imodv03	0.55	0.39	0.07	0.44	0.69	0.40	0.24
	md	0.55	0.38	0.07	0.46	0.68	0.41	0.28
	NOLB	0.53	0.39	0.08	0.43	0.66	0.40	0.23

*Selected prediction threshold $\Delta\Delta G = 0.45$ based on validation three-state Macro F1.

Table S10. Three-state classification performance on the original (non-augmented) S461 dataset (direct). Destabilizing if $\Delta\Delta G \leq -1$ kcal/mol, stabilizing if $\Delta\Delta G \geq 1$ kcal/mol, otherwise neutral. Reported are strict accuracy (Sc), off-by-one (Of1), off-by-two (Of2), Macro F1, and class-wise F1 scores.

**Method**	**Sc**	**Of1**	**Of2**	**Macro F1**	**F1 Destab**	**F1 Neutral**	**F1 Stab**
FoldX	0.63	0.32	0.05	0.49	0.63	0.69	0.15
MAESTRO	0.72	0.28	0.00	0.48	0.71	0.74	0.00
PremPS	0.69	0.31	0.00	0.52	0.66	0.73	0.15
Dynamut	0.65	0.34	0.01	0.48	0.56	0.72	0.17
mCSM	0.69	0.30	0.00	0.47	0.70	0.71	0.00
SDM	0.66	0.33	0.01	0.51	0.62	0.71	0.19
DUET	0.72	0.27	0.00	0.49	0.72	0.75	0.00
I-Mutant3.0	0.66	0.34	0.01	0.44	0.64	0.69	0.00
DDGun3D	**0.73**	0.26	0.01	**0.56**	0.71	**0.77**	**0.20**
ACDC-NN	**0.73**	0.27	0.00	0.54	0.69	**0.77**	0.15
INPS3D	0.70	0.30	0.01	0.47	0.70	0.71	0.00
PoPMuSiC	0.72	0.28	0.00	0.53	0.72	0.73	0.14
ThermoNet	0.68	0.31	0.01	0.49	0.60	0.75	0.11
RaSP	**0.73**	0.26	0.01	0.50	**0.74**	0.75	0.00
OrgNet	0.69	0.31	0.00	0.46	0.64	0.74	0.00
OrgNet^{+}	0.68	0.31	0.01	0.52	0.66	0.72	0.19
**OrgNet**$^{+*}$	0.66	0.32	0.01	0.51	0.68	0.68	0.17

* Selected prediction threshold $\Delta\Delta G = 0.75$ based on validation Macro F1.

Table S11. Three-state classification performance on the original (non-augmented) S461 dataset (direct). Destabilizing if $\Delta\Delta G \leq -0.5$ kcal/mol, stabilizing if $\Delta\Delta G \geq 0.5$ kcal/mol, otherwise neutral. Reported are strict accuracy (Sc), off-by-one (Of1), off-by-two (Of2), Macro F1, and class-wise F1 scores.

**Method**	**Sc**	**Of1**	**Of2**	**Macro F1**	**F1 Destab**	**F1 Neutral**	**F1 Stab**
FoldX	0.60	0.29	0.11	0.47	0.70	0.54	0.16
MAESTRO	0.65	0.33	0.02	0.46	0.76	0.52	0.09
PremPS	0.67	0.32	0.01	0.49	0.76	0.56	0.14
Dynamut	0.58	0.35	0.06	0.45	0.66	0.56	0.13
mCSM	0.67	0.30	0.03	0.42	0.78	0.49	0.00
SDM	0.56	0.36	0.08	0.47	0.67	0.50	0.23
DUET	0.68	0.29	0.03	0.47	0.77	0.56	0.06
I-Mutant3.0	0.65	0.32	0.03	0.41	0.76	0.48	0.00
DDGun3D	0.63	0.33	0.04	0.50	0.72	0.54	0.22
ACDC-NN	0.64	0.34	0.02	0.47	0.72	0.57	0.13
INPS3D	0.68	0.30	0.02	0.46	0.78	0.52	0.07
PoPMuSiC	0.68	0.30	0.02	0.46	0.79	0.53	0.06
ThermoNet	0.56	0.42	0.02	0.44	0.62	0.52	0.17
RaSP	0.68	0.29	0.03	0.48	0.78	0.53	0.13
OrgNet	0.60	0.37	0.03	0.44	0.70	0.52	0.10
OrgNet^{+}	0.62	0.36	0.03	0.49	0.72	0.48	0.26
**OrgNet**$^{+*}$	0.62	0.36	0.03	0.49	0.73	0.45	0.29

* Selected prediction threshold $\Delta\Delta G = 0.45$ based on validation Macro F1.

Table S12. Three-state classification performance on the original (non-augmented) S669 dataset (direct). Destabilizing if $\Delta\Delta G \leq -1$ kcal/mol, stabilizing if $\Delta\Delta G \geq 1$ kcal/mol, otherwise neutral. Reported are strict accuracy (Sc), off-by-one (Of1), off-by-two (Of2), Macro F1, and class-wise F1 scores.

Method	Sc	Of1	Of2	Macro F1	F1 Destab	F1 Neutral	F1 Stab
FoldX	0.56	0.38	0.06	0.45	0.58	0.61	0.16
MAESTRO	0.62	0.36	0.01	0.43	0.62	0.67	0.00
PremPS	0.61	0.37	0.02	0.43	0.59	0.66	0.03
Dynamut	0.57	0.42	0.02	0.43	0.48	0.66	0.15
mCSM	0.60	0.37	0.03	0.42	0.63	0.63	0.00
SDM	0.56	0.41	0.03	0.45	0.53	0.63	0.19
DUET	0.61	0.37	0.02	0.43	0.63	0.65	0.00
I-Mutant3.0	0.59	0.39	0.03	0.41	0.58	0.64	0.00
DDGun3D	0.63	0.34	0.02	0.50	0.63	0.69	0.18
ACDC-NN	0.64	0.35	0.01	0.46	0.60	0.71	0.07
INPS3D	0.61	0.37	0.03	0.44	0.62	0.64	0.07
PoPMuSiC	0.62	0.36	0.03	0.44	0.63	0.65	0.04
ThermoNet	0.59	0.40	0.01	0.41	0.50	0.68	0.06
RaSP	0.63	0.34	0.03	0.44	0.65	0.67	0.00
OrgNet	0.60	0.39	0.01	0.44	0.54	0.67	0.12
OrgNet$^+$	0.60	0.39	0.02	0.44	0.58	0.66	0.09
**OrgNet**$^{+*}$	0.57	0.40	0.02	0.44	0.60	0.60	0.10

* Selected prediction threshold $\Delta\Delta G = 0.75$ based on validation Macro F1.

Table S13. Three-state classification performance on the original (non-augmented) S669 dataset (direct) using $\Delta\Delta G$ threshold 0.5 kcal/mol. Destabilizing if $\Delta\Delta G \leq -0.5$ kcal/mol, stabilizing if $\Delta\Delta G \geq 0.5$ kcal/mol, otherwise neutral. Reported are strict accuracy (Sc), off-by-one (Of1), off-by-two (Of2), Macro F1, and class-wise F1 scores.

Method	Sc	Of1	Of2	Macro F1	F1 Destab	F1 Neutral	F1 Stab
FoldX	0.54	0.32	0.14	0.46	0.65	0.46	0.26
MAESTRO	0.56	0.38	0.06	0.42	0.70	0.45	0.10
PremPS	0.58	0.36	0.06	0.44	0.70	0.48	0.14
Dynamut	0.51	0.41	0.08	0.44	0.59	0.48	0.26
mCSM	0.60	0.31	0.09	0.40	0.73	0.42	0.04
SDM	0.51	0.38	0.11	0.45	0.62	0.45	0.28
DUET	0.60	0.32	0.08	0.44	0.73	0.49	0.09
I-Mutant3.0	0.57	0.35	0.08	0.38	0.71	0.42	0.00
DDGun3D	0.55	0.37	0.07	0.47	0.67	0.47	0.27
ACDC-NN	0.55	0.41	0.04	0.43	0.66	0.50	0.12
INPS3D	0.60	0.33	0.07	0.43	0.73	0.45	0.12
PoPMuSiC	0.59	0.34	0.08	0.41	0.72	0.44	0.08
ThermoNet	0.48	0.48	0.04	0.40	0.57	0.46	0.15
RaSP	0.59	0.34	0.07	0.44	0.72	0.45	0.16
OrgNet	0.53	0.40	0.07	0.44	0.64	0.46	0.22
OrgNet$^+$	0.53	0.42	0.05	0.44	0.67	0.39	0.24
**OrgNet**$^{+*}$	0.55	0.40	0.05	0.45	0.69	0.37	0.30

* Selected prediction threshold $\Delta\Delta G = 0.45$ based on validation Macro F1.

Table S14. Performance of OrgNet and OrgNet$^+$ on the augmented S669 and S461 datasets (Direct), where mutations are classified into buried (< 0.2) and surface (≥ 0.2) categories based on solvent accessible surface area (SASA). Reported are prediction accuracy metrics (RMSE, MAE, Pearson r) and prediction uncertainty (σ_{pred}). Best values are highlighted in bold.

Dataset	Mutation class	Method	N	RMSE	MAE	r	σ_{pred}
S669	buried (SASA < 0.2)	OrgNet	127673	1.82	1.39	0.31	0.722
		OrgNet$^+$		**1.63**	**1.24**	**0.43**	**0.496**
	surface (SASA ≥ 0.2)	OrgNet	198297	1.55	1.12	0.20	0.469
		OrgNet$^+$		**1.47**	**1.05**	**0.29**	**0.368**
S461	buried (SASA < 0.2)	OrgNet	87085	1.58	1.24	0.40	0.767
		OrgNet$^+$		**1.29**	**1.01**	**0.55**	**0.517**
	surface (SASA ≥ 0.2)	OrgNet	142166	1.13	0.85	0.27	0.460
		OrgNet$^+$		**1.06**	**0.79**	**0.33**	**0.357**

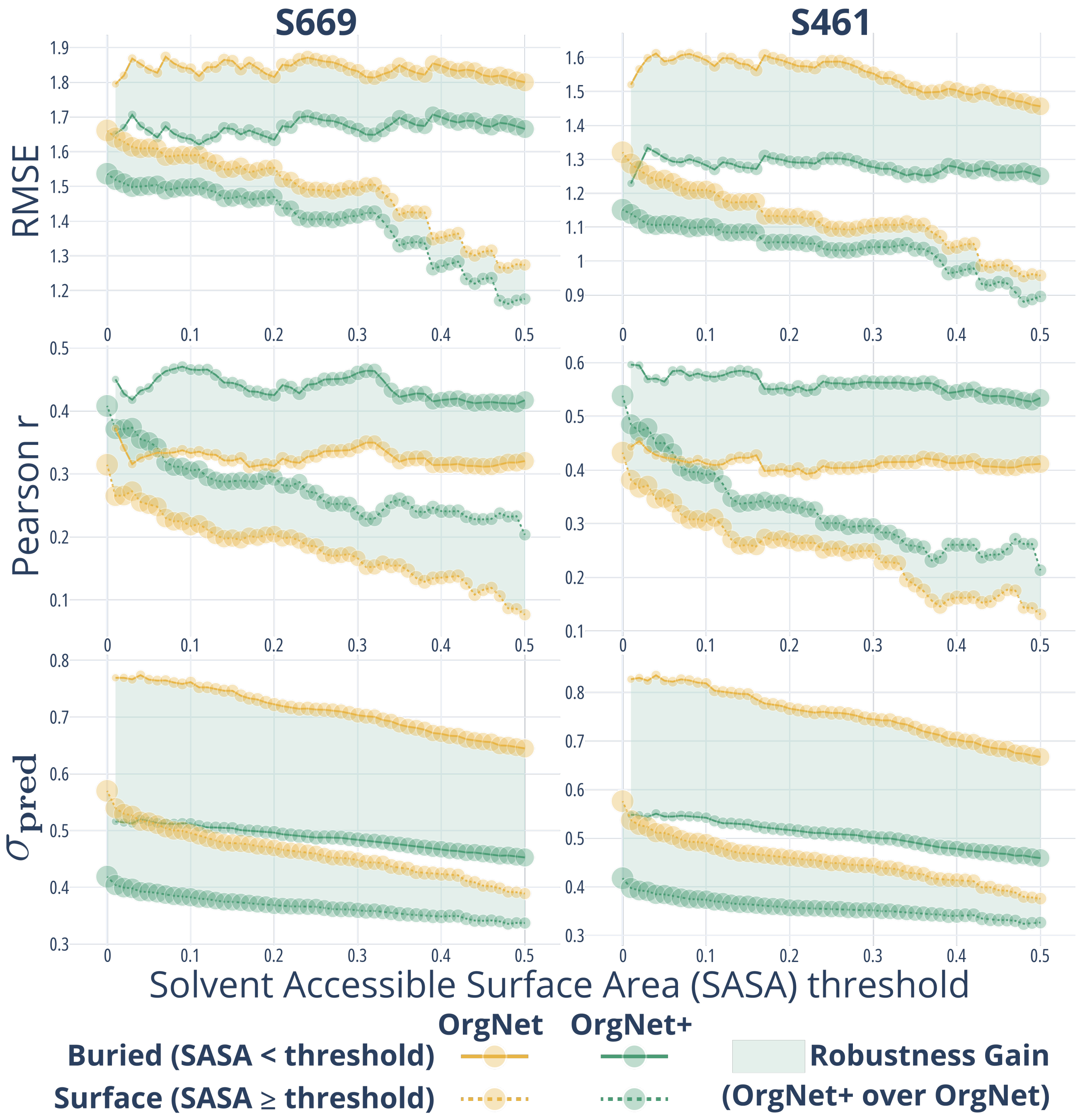

Fig. S2. Point mutations in the augmented S669 and S461 datasets (Direct) are classified into buried and surface categories based on the solvent accessible surface area (SASA) of the wild-type residue in the reference protein structures. For each threshold, point mutations are partitioned into buried (SASA $<$ threshold) and surface (SASA $\geq$ threshold) subsets, and prediction performance is evaluated separately for each subset. The point at threshold $= 0$ corresponds to the full dataset (i.e., no partitioning), and is included for reference. Shown are RMSE, Pearson correlation coefficient (r), and prediction uncertainty (σ_{pred}), computed for both OrgNet and OrgNet^{+}. Marker size reflects the number of point mutations in the corresponding subset, with counts for buried and surface mutations summing to the total dataset size at each threshold.

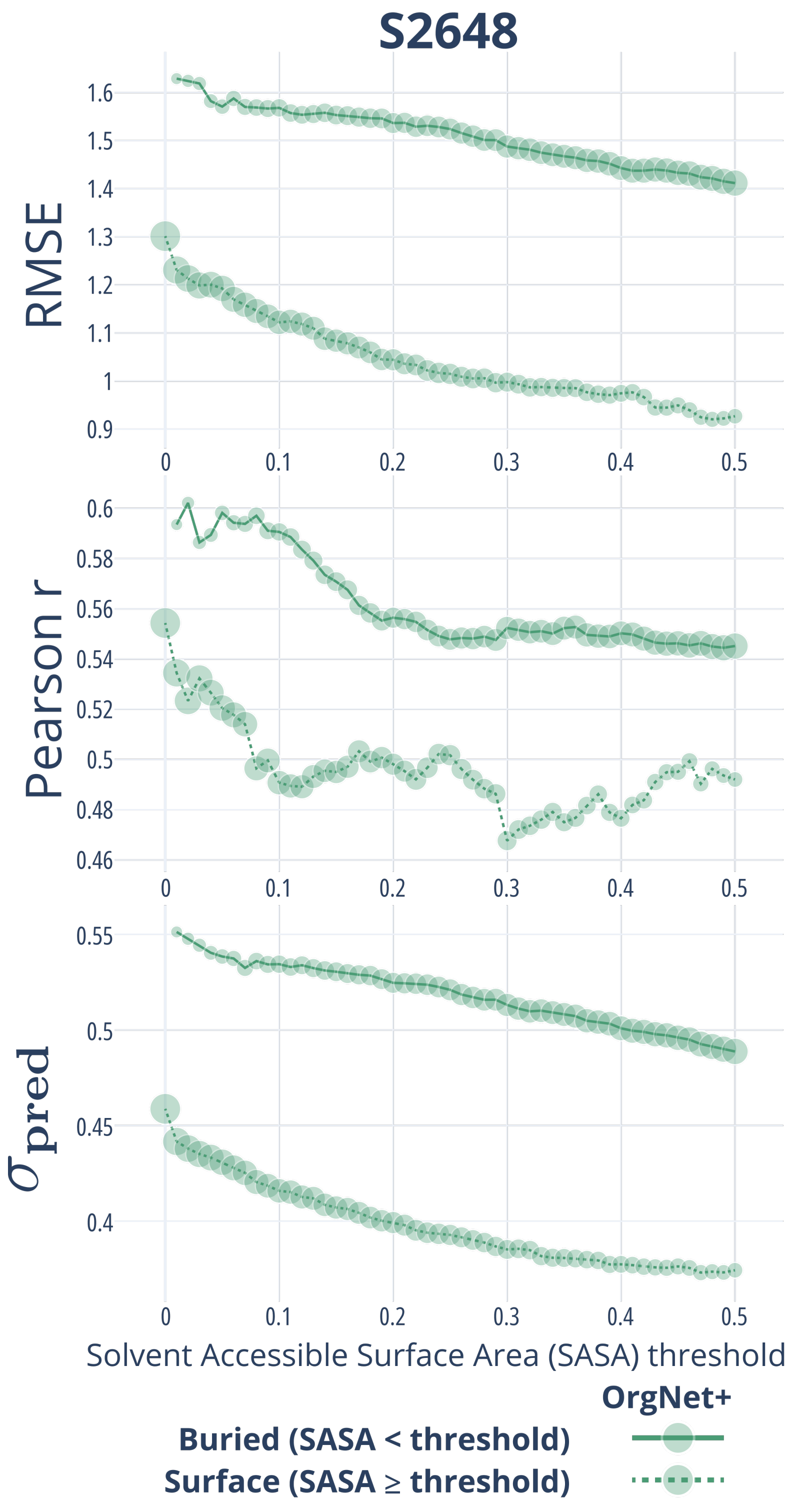

Fig. S3. Point mutations in the augmented OrgNet^{+} training dataset S2648 (Direct) are classified into buried and surface categories based on the solvent accessible surface area (SASA) of the wild-type residue in the reference protein structures. For each threshold, mutations are partitioned into buried (SASA $<$ threshold) and surface (SASA $\geq$ threshold) subsets, and prediction performance is provided separately for each subset. The point at threshold $= 0$ corresponds to the full dataset (i.e., no partitioning) and is included for reference. Shown are RMSE, Pearson correlation coefficient (r), and prediction uncertainty (σ_{pred}). Marker size reflects the number of mutations in each subset, with counts for buried and surface mutations summing to the total dataset size at each threshold.
